# Exosome-Coated Prussian Blue Nanoparticles for Specific Targeting and Treatment of Glioblastoma

**DOI:** 10.1021/acsami.4c02364

**Published:** 2024-04-10

**Authors:** Meghan L. Hill, Seock-Jin Chung, Hyun-Joo Woo, Cho Rong Park, Kay Hadrick, Md Nafiujjaman, Panangattukara Prabhakaran Praveen Kumar, Leila Mwangi, Rachna Parikh, Taeho Kim

**Affiliations:** ^1^Department of Biomedical Engineering, ^2^Department of Chemical Engineering and Materials Science, ^3^Department of Human Biology, Lyman Briggs Honors College, and ^4^Institute for Quantitative Health Science and Engineering, Michigan State University, East Lansing, Michigan 48824, United States

**Keywords:** theranostics, molecular imaging, glioblastoma, exosome, Prussian blue nanoparticles, photoacoustic imaging, photothermal therapy

## Abstract

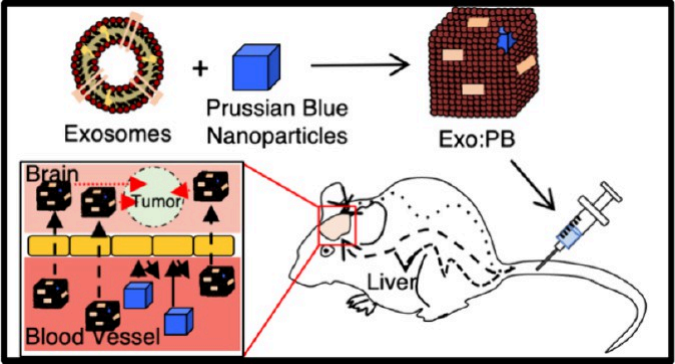

Glioblastoma is one of the most aggressive and invasive types of brain cancer with a 5-year survival rate of 6.8%. With limited options, patients often have poor quality of life and are moved to palliative care after diagnosis. As a result, there is an extreme need for a novel theranostic method that allows for early diagnosis and noninvasive treatment as current peptide-based delivery standards may have off-target effects. Prussian Blue nanoparticles (PBNPs) have recently been investigated as photoacoustic imaging (PAI) and photothermal ablation agents. However, due to their inability to cross the blood–brain barrier (BBB), their use in glioblastoma treatment is limited. By utilizing a hybrid, biomimetic nanoparticle composed of a PBNP interior and a U-87 cancer cell-derived exosome coating (Exo:PB), we show tumor-specific targeting within the brain and selective thermal therapy potential due to the strong photoconversion abilities. Particle characterization was carried out and showed a complete coating around the PBNPs that contains exosome markers. *In vitro* cellular uptake patterns are similar to native U-87 exosomes and when exposed to an 808 nm laser, show localized cell death within the specified region. After intravenous injection of Exo:PB into subcutaneously implanted glioblastoma mice, they have shown effective targeting and eradication of tumor volume compared to PEG-coated PBNPs (PEG:PB). Through systemic administration of Exo:PB particles into orthotopic glioblastoma-bearing mice, the PBNP signal was detected in the brain tumor region through PAI. It was seen that Exo:PB had preferential tumor accumulation with less off-targeting compared to the RGD:PB control. *Ex vivo* analysis validated specific targeting with a direct overlay of Exo:PB with the tumor by both H&E staining and Ki67 labeling. Overall, we have developed a novel biomimetic material that can naturally cross the BBB and act as a theranostic agent for systemic targeting of glioblastoma tissue and photothermal therapeutic effect.

## Introduction

Glioblastoma is the most aggressive type of primary brain cancer that has a 5-year survival rate of 6.8%.^1^ Common symptoms include headaches, problems with speech, memory loss, confusion, and vision problems.^2,3^ Due to the overlapping nature of these symptoms with many other neurological disorders, glioblastoma can be extremely difficult to diagnose without expensive imaging and invasive procedures. In addition, diagnostic procedures are painful for the patient to undergo before starting therapy. Even after diagnosis, there are few chemotherapy or radiation treatment options available to the patient due to the impenetrable nature of the blood–brain barrier (BBB).^4−6^ Unfortunately, this often leads to palliative care for patients diagnosed with glioblastoma.

Nanoparticles have become a novel approach for diagnostics and therapeutics in brain-related diseases.^6,7^ Prussian Blue nanoparticles (PBNPs) are FDA-approved agents for treating radiation exposure.^8^ They work by absorbing heavy metals into their cubic matrix, allowing for efficient disposal through the body.^9^ With an intense light absorbance in the biological transparency window (peak absorbance between 700 and 750 nm), their use in disease-related phototheranostics is promising. The near-infrared (NIR) light absorption of PBNPs is attributed to the intervalence charge-transfer band (Fe^2+^/Fe^3+^).^10^ This makes them ideal candidates for NIR light-based imaging or therapeutic applications. As aggregation can be a primary problem of nanoparticle-based therapies, facile synthesis of PBNPs using citric acid as a stabilizer can easily prevent this issue.^4,11^ The carboxylic groups form ionic bonds with the Fe^2+^/Fe^3+^ valences on the particle surfaces, which create a stable surfactant structure.^12^ This can also make it easy for further surface modification with specific targeting moieties through chemical conjugation.^4,11,13,14^

The traditional method of diagnosing brain cancer is using magnetic resonance imaging (MRI).^14−17^ MRI has superior soft tissue contrast and can easily distinguish cancerous tumors from healthy tissue, especially when coupled with iron oxide, gadolinium-based agents, or newer manganese-containing contrast agents.^17,18^ Unfortunately, due to long acquisition times and high cost, it is difficult to use for constant monitoring of tumor growth or therapeutics. Photoacoustic imaging (PAI) is an emerging imaging modality that can bridge this gap as it benefits from both optical and acoustic imaging sources. Based on the “light in–sound out” approach, tissue is exposed to a pulsed laser light that causes local thermoelastic expansion.^19^ This expansion causes small acoustic waves to be produced within the surrounding medium that can then be relayed to an external transducer, which produces an image.^14,20^ This imaging technique has excellent spatial and temporal resolution and is currently in the early stages of clinical translation. PAI is great for real-time detection and diagnosis of brain-related diseases, including brain cancer, due to its advantages in deep-tissue penetration of ultrasound imaging and high resolution of optical signatures.^14,21,22^ These signatures are obtained using a pulsed laser light between 680 and 850 nm that allows for hemoglobin, deoxyhemoglobin, and exogenous agent PAI signals to be seen. PBNPs can act as powerful exogenous contrast agents for PAI by absorbing outside the Hb and HbO_2_ windows and can help identify target tissues such as tumor regions.^23^

Photothermal therapy (PTT) is a minimally invasive treatment strategy often used for cancers.^24^ Particularly for brain cancers, PTT may reduce surgical risks associated with open brain surgery (e.g., craniotomy) and is easy to apply on an outpatient basis.^16,25,26^ Photosensitizing materials with high light-to-heat conversion capabilities upon NIR light irradiation are often required to augment PTT efficacy. Gold nanorods (AuNRs) are the most widely used nanoparticles for photothermal application.^27^ Unfortunately, AuNRs suffer from photodegradation, and so when exposed to laser light multiple times, they will start to deform into nanospheres and are no longer useful for therapy. However, PBNPs have high photostability and are extremely biocompatible.^4,13,23,28^ They have particularly been effective for enhanced localized cell death of tumor cells and have shown little to no off-target effects. Furthermore, while PTT is known to cause local inflammation with excessive ROS presence, PBNPs can act as enzymatic scavengers based on alternating Fe^2+^/Fe^3+^ surface valence, which can lower the PTT side-effects.^10,29,30^ Overall, PBNPs can serve as great PTT agents that can increase treatment efficacy. To date, PBNPs have not been used in the diagnosis or treatment of brain cancers due to their inability to cross the BBB. Their current uses are limited to photothermal therapy and photoacoustic identification of mostly superficial cancers.^31,32^

Unfortunately, many nanoparticles suffer from the same limitations as chemotherapies for brain tumor delivery, as they cannot pass through the BBB.^5,6,33^ The most common way to circumvent this problem is through external disruption of the BBB using focused ultrasound with circulating microbubbles.^34^ While this can be very effective, it also causes a lot of damage to the barrier, which might allow other toxins or foreign substances to enter the brain region. Another popular delivery method is the use of conjugated peptides such as RGD, cyclic RGD, and PL3 to target glioblastoma due to overexpression of recognizing integrins on the surface of cancer cells.^35^ With many of the current FDA clinical trial studies including RGD (cilengitide) as the surface moiety, the peptide–nanoparticle conjugates are the standard to be matched for clinical translation.^36^ However, one of the main problems for peptide delivery is off-target effects that can occur based on the expression of surface integrins that recognize these different sequences on healthy cells.^37^ This leads to the dilemma of premiere active targeting in cancerous cells with slight accumulation in normal tissue depending on which the peptide encounters first. Extracellular vesicles, particularly exosomes (70–120 nm), are known to have the innate ability to pass through the BBB. Exosomes expressing tetraspanin proteins, such as CD63, CD81, CD9, and flotillin-1, help transfer of therapeutics to the brain parenchyma.^38−41^ This is specifically done through receptor-mediated phagocytosis. As exosomes pass through the brain microvessel endothelial cells, they are transported in multivesicular bodies (MVBs). During this stage, lysosomes will discard foreign material within the MVBs, but the exosomes are further transported to the brain due to the expression of tetraspanin proteins.^42^ Based on the originating cell line, the exosomes can be used as accurate active targeting moieties and thus have great potential for cancer detection and drug delivery.^43^ Utilizing U-87 derived exosomes takes advantage of this effect and allows us to specifically target the U-87 induced tumors that we used within our animal models. PBNPs typically have similar size distributions to exosomes, so using mechanical force, we could easily create hybrid particles with increased stability that retain the innate abilities of both particles. With the nanohybrids, systemic brain tumor targeting and efficient phototheranostics for glioblastoma can be achieved, such as in tumor resection or laser interstitial thermal therapy (LITT).^44−46^

**Figure 1:**
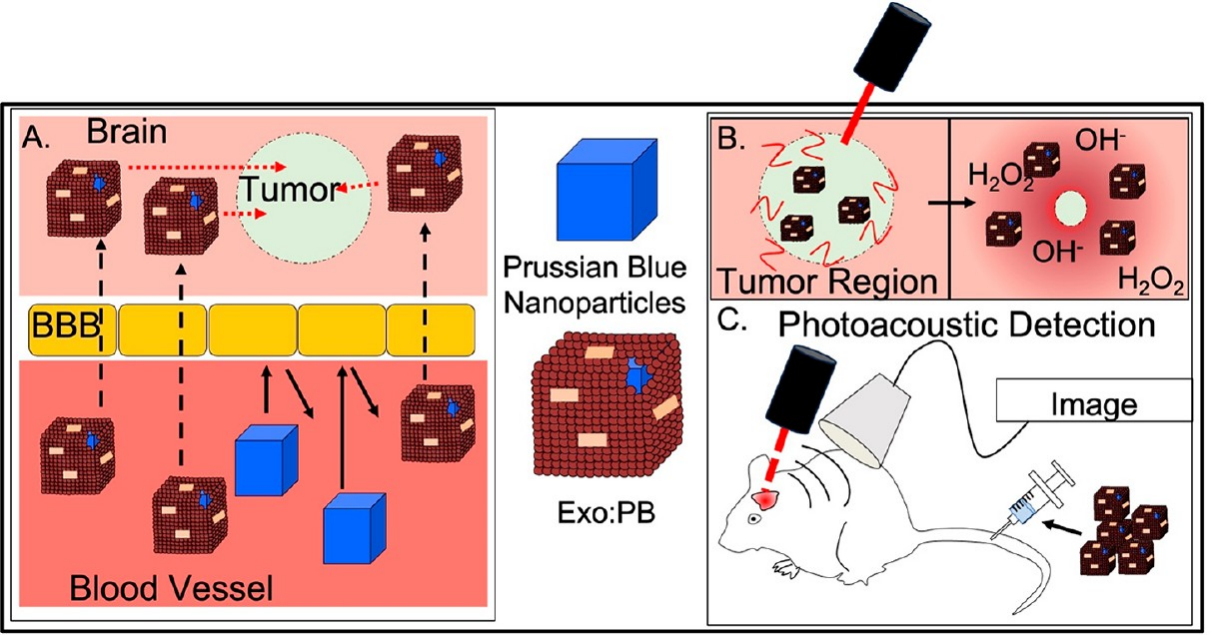
Graphical representation of Exo:PB targeting potential, therapeutic effect, and diagnostic ability. (A) After iv injection, hybrid Exo:PB particles are able to pass through the blood–brain barrier (BBB) and accumulate within the tumor region, unlike the uncoated PBNPs. (B) Once particles are exposed to a continuous-wave NIR laser excitation, they cause a local photothermal ablation effect to reduce the tumor mass and help scavenge ROS species present after treatment. (C) Photoacoustic detection of the brain tumor region after iv injection of Exo:PB particles. A pulsed laser light excites the particles/tissue within the exposed region, which causes thermoelastic expansion that is picked up by an external transducer, producing an image.

In this paper, we demonstrate utilizing U-87-derived exosomes as a coating for PBNPs (Exo:PB) to specifically target and treat glioblastoma tumors (Figure 1). We show increased glioma targeting of the nanohybids using *in vivo* imaging. PBNPs enhanced PAI contrast of brain tumors with consistent signal for up to 24 h postinjection. After exposure to an NIR laser, tumor size is shown to shrink, and *ex vivo* analysis validates localized apoptotic death. Overall, we present a novel noninvasive method to detect and treat orthotopic glioblastoma tumors using hybrid Exo:PB particles.

## Results and Discussion

### Synthesis and Characterization of Prussian Blue Based Nanoparticles

**Figure 2:**
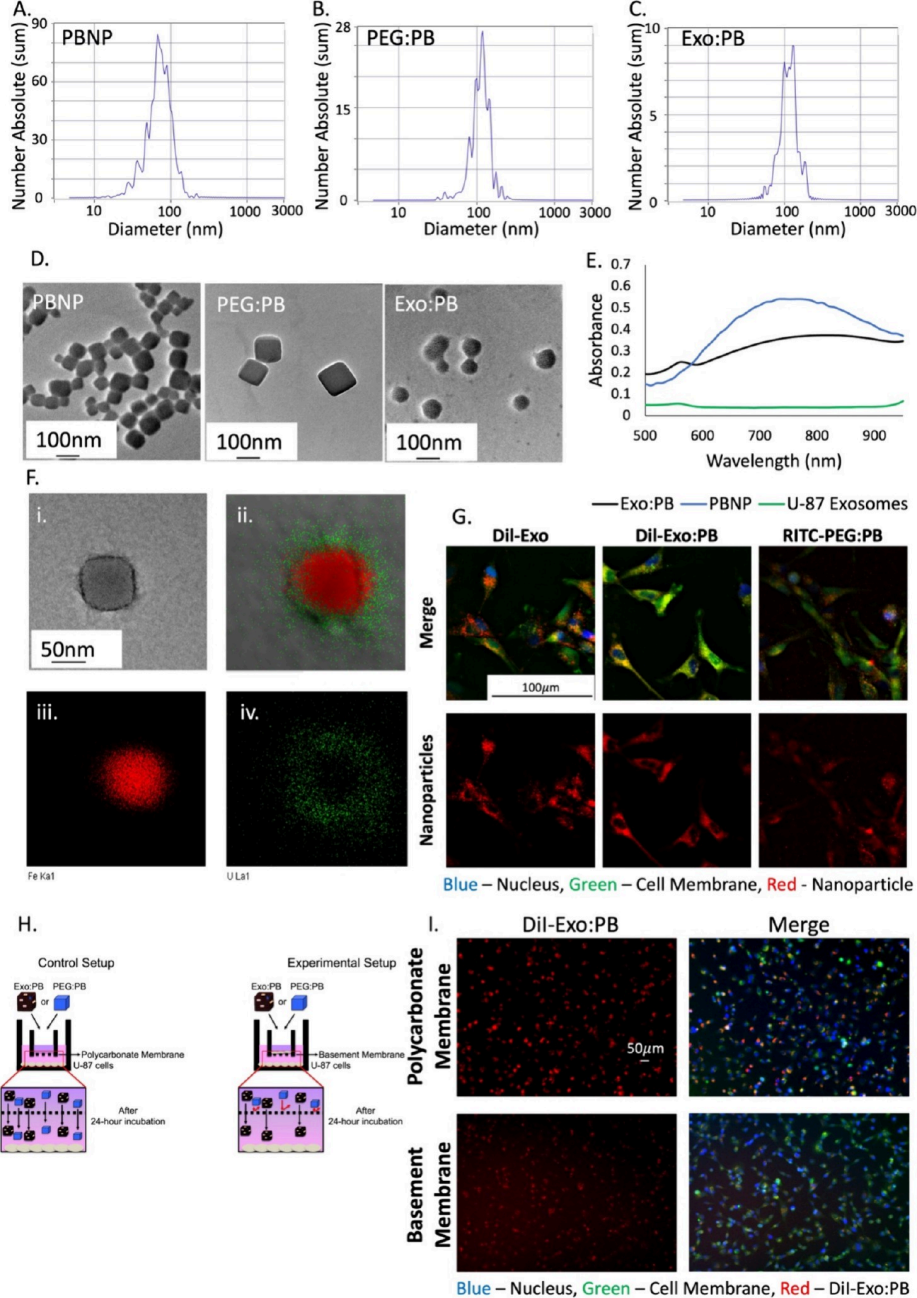
Particle and exosome characterization. NTA size distribution of (A) PBNP, (B) PEG:PB, and (C) Exo:PB. (D) TEM images of PBNP, PEG:PB, and Exo:PB particles. Scale bar is 100 nm. (E) Absorbance of PBNP (blue), Exo:PB (black), and U-87 exosomes (green). (F) (i, ii) High-magnification TEM image of uranyl acetate-stained Exo:PB (green = uranium signal, red = iron signal); (iii) iron electron mapping signal; (iv) uranium electron mapping signal. Scale bar is 50 nm. (G) Cell uptake patterns of DiI-Exosomes, DiI-Exo:PB, and RITC-PEG:PB particles in U-87 cells. Red = nanoparticle signal, blue = cell nucleus, green = cell membrane. Scale bar is 100 μm. (H) Schematic of *in vitro* BBB setup and expected results. (I) *In vitro* BBB uptake patterns for DiI-Exo:PB particles in U-87 cells. Blue = cell nucleus, green = cell membrane, red = DiI-Exo:PB. Scale bar = 50 μm.

PBNPs were synthesized using a simple co-precipitation method using FeCl_3_ and K_4_[Fe(CN)_6_] in the presence of citric acid. Standard reaction size was scalable up to 0.15 g, maintaining an average particle size of ∼70 nm and ζ potential of −42.1 mV as verified through nanoparticle tracking analysis (NTA) and dynamic light scattering (DLS) (Figures 2A and S1A). Particle morphology was shown to be cubic (Figure 2D). Citric acid was chosen as the surfactant to prevent aggregation. Functionalization with polyethylene glycol (PEG) of the particle surface was done using polyvinyl pyrrolidone (PVP) and NH_2_-PEG-NH_2_ substitution. The initial PVP coating allows for passive conjugation of the NH_2_-PEG-NH_2_ through a basic hydrolysis substitution in ethanol, in which the PVP will detach from Fe^3+^ ions and allow for the NH_2_ groups of the PEG to bond without direct competition with OH^–^ ions in solution. The conjugation was verified using Fourier transform infrared spectroscopy (FTIR), in which peaks for NH were seen in the same profile as the PBNP CN marker (Figure S2A). After PEGylation, size and ζ potential of the particle were shown to change to ∼100 nm and −9.54 mV (Figures 2B and S1A). Transmission electron microscopy (TEM) results show a consistent cubic shape and good dispersity (Figure 2D). Further surface modification was done to conjugate RGD peptide to the surface of citrate-capped PBNPs. Initially, a RITC-RGD conjugate was formed using a thiourea reaction (Figure S3A). Calculated molecular weight with a chemical formula of C_41_H_52_N_9_O_9_S (M + H^+^) was 846.3603 g/mol. Through mass spectrometry analysis, the RITC-RGD compound was validated with a molecular weight of 846.3606 g/mol (Figure S3B). RITC-RGD was conjugated to PBNPs through a hydrolysis reaction between the open OH groups of RGD and citric acid. Particle conjugation was verified using FTIR analysis with the presence of NH and C=C peaks for RGD and RITC as well as CN for PBNP (Figure S2B). Once the particles were purified, particle morphology was cubic with an average size of ∼80 nm (Figure S4A,B) and had a peak absorbance between 700 and 750 nm (Figures S4C). The amount of RGD present on the surface of the RGD:PB particles was determined to be 0.3 mg/mL based on a fluorescamine assay (Figure S4D).

To create the Exo:PB nanohybrids, the cubic-shaped PBNPs were mixed with U-87 derived exosomes, which were isolated using a differential ultracentrifugation method and then extruded through a 200 nm membrane (Figure S5). Through the extrusion process, the color shifted from transparent light blue to a darker opaque blue, indicating the coating was successful. There is also an evident size shift of ∼70 nm to ∼120 nm from PBNP and U-87 exosome to Exo:PB (Figures 2C and S1A,D) with a more rounded appearance of particles after extrusion (Figure 2D). While differences in size and morphology are good indicators of successful coating, extra validation was done using Western blotting and electron mapping to ensure a full and even layer over the PBNP that still contains typical exosome markers (Figure S1C). Using uranyl acetate, the exosome layer of Exo:PB particles could be stained and detected based on the uranium signal. It was seen through electron mapping that the particles contain an iron core and are coated with an even uranium layer (exosome) (Figures 2F and S6). Coupled with Western blotting results that show the presence of Flotillin-1 in both the U-87 derived exosome and Exo:PB particles (Figure S1C), we find that the Exo:PB particles contain a PBNP core with a successfully coated exosome layer. After extrusion, the particles are shown to be stable for up to nine months at 4 °C (Figure S1B), which is good for possible translational efforts. Previous studies have reported iron oxide-extracellular vesicle hybrids prepared through chemical conjugation, electroporation, incubation, and extrusion methods. However, these hybrid particles have shown to suffer from short-term stability and destruction of the EV layer over time.^47,48^ Cellular uptake patterns of Exo:PB particles are also shown to be similar to that of native DiI-stained U-87 exosomes as similar fluorescent signal is detected intracellularly in the perinuclear regions after 1 h of incubation (Figure 2G). In comparison, the RITC-PEG:PB fluorescent signal is not as prevalent, indicating our Exo:PB particles led to a higher cellular internalization through membrane fusion, receptor mediated uptake and endocytosis.^49^ Exo:PB cellular uptake kinetics were evaluated and showed maximum concentration within U-87 cells between 7 and 8 h (Figure S7). This is important information to determine dose for injection *in vivo*. It can also help determine the best time point to perform photothermal therapy in which the best photoablation effects can be seen. To test the efficacy of Exo:PB particles vs PEG:PB and RGD:PB for use in brain tumor targeting and therapeutics, cells were added to well-inserts containing either a porous polycarbonate membrane or a basement membrane that mimics the BBB with U-87 cells plated on the bottom of the well (Figure 2H). After 24 h, all of DiI-Exo:PB, RITC-PEG:PB, and RITC-RGD:PB showed normal uptake patterns with the control wells containing the polycarbonate membrane, but only DiI-Exo:PB and RITC-RGD:PB showed the capability to pass through the basement membrane matrix with reduced signal (Figures 2I and S8). Tumorigenesis of U-87 cells after exposure to U-87 exosomes or Exo:PB was investigated by looking at the growth rate of cells 24 and 48 h postincubation. Statistically, there was no difference in growth in comparison to nontreated cells (Figure S9). This indicates that there is not a direct cause of cancerous growth due to exosome exposure.

### Photothermal Capabilities

**Figure 3:**
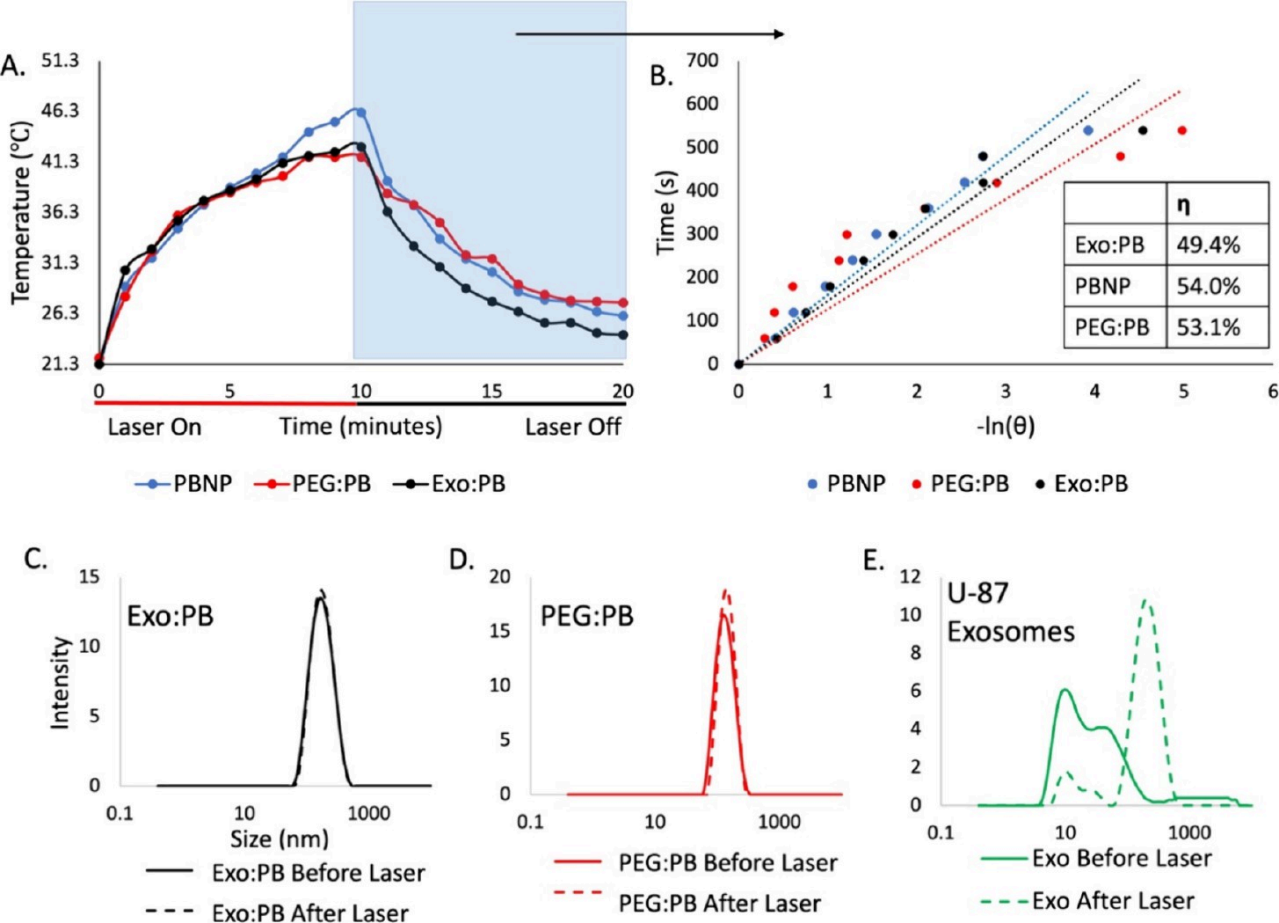
Laser exposure effect on particles. (A) Temperature scaling of Exo:PB (black), PEG:PB (red), and PBNP (blue) when exposed to an 808 nm laser on (red) and off (black). (B) Linear distribution of the cooling curve of (A) highlighted in blue. Blue = PBNP, red = PEG:PB, and black = Exo:PB. Table indicates calculated photothermal conversion efficiencies (η). DLS size distribution of (C) Exo:PB, (D) PEG:PB, and (E) U-87 exosomes before and after 1 min exposure to an 808 nm laser.

PBNPs have a characteristic light absorbance within the biological transparency window, as defined by their peak absorbance between 700 and 750 nm (Figure 2E). Due to the ability of PBNPs to transfer light energy from a laser into heat, they can be used for photothermal therapy. Unlike AuNRs that show a decrease in photoconversion ability due to particle morphological changes (Figure S10), PBNPs maintain great photostability upon repeated laser exposure. To validate the robust photothermal capabilities, 0.5 mg/mL PBNPs were exposed to a laser (808 nm, 2 W/cm^2^) in multiple 10 min time increments and allowed to cool back to room temperature. The PBNP-containing particles reached increasingly higher temperatures after each respective cool-down period (Figure S10), with maximum temperatures of 52 °C for PBNP. When PEG:PB and Exo:PB were exposed to the same laser conditions for 10 min, the maximum temperature reached was 41.8 °C for PEG (PEG:PB) and 42.8 °C for exosome-coated PBNPs (Exo:PB), respectively (Figures 3A and S11). The difference in the maximum temperature reached is likely due to the external coating of either PEG or exosome absorbing a fraction of the light energy. The calculated photothermal conversion efficiencies (η) for PBNP, PEG:PB, and Exo:PB are similar at 54.0%, 53.1%, and 49.4% respectively (Figure 3B). This can be confirmed by the size change of U-87 exosomes after exposure to a laser (Figure 3C–E). Lipid-based particles are known to have long-term stability problems due to hydrolysis and oxidation of lipids that either occurs naturally or by an outside energy source.^50^ Based on the generation of various radicals in the solution such as CH_3_, CO, or CHO, these particles rapidly degrade and reform into bigger vesicles.^51^ Interestingly, there appears to be no separate population in the Exo:PB, which indicates the exosome membrane on the PBNPs stays intact throughout the photoconversion process. The robust coating, as well as the catalytically active PBNP surface chemistry, may contribute to strong membrane stability.^52^

**Figure 4:**
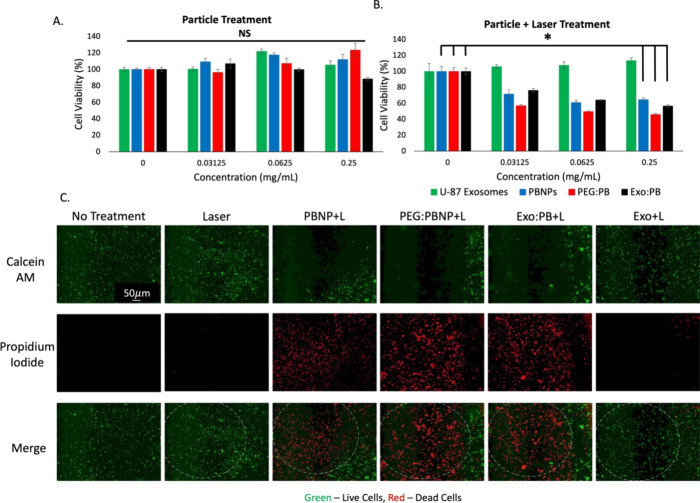
*In vitro* cytotoxicity and photothermal ablation effects. (A) MTT assay of U-87 exosomes (green), PBNPs (blue), PEG:PB (red), and Exo:PB (black). (B) MTT assay of U-87 exosomes (green), PBNPs (blue), PEG:PB (red), and Exo:PB (black) after 1 min exposure to an 808 nm laser. *p < 0.05, NS = no significance. 0 mg/mL concentration refers to no particles added. (C) Live and dead cell assay results of U-87 cells incubated with PBNP, PEG:PB, Exo:PB, and U-87 exosomes and exposed to an 808 nm laser. Green = live cells, red = dead cells. Scale bar = 50 μm.

To further validate the photothermal therapy potential of these particles, we treated the particles *in vitro* against a U-87 cell line. Through MTT assay, we can see no toxicity of particles up to 0.25 mg/mL, but once exposed to the 808 nm laser, cell viability is reduced by nearly 50% in wells containing PBNP-based particles (Figure 4A,B). These results prove that the Exo:PB particles could be useful in PTT. A live and dead cell assay (calcein AM/propidium iodide staining) was done next to study localized cell death upon laser treatment with PB particles. When treated cells were imaged by fluorescence microscopy, in areas where the laser was exposed, there was a distinct area of cell death (as indicated in red), but areas where there was no laser showed healthy cells (as indicated in green) (Figure 4C). To investigate the maximum temperature reached on the cellular level to validate photothermal effects from the particles, the overall temperature gain from the treated U-87 cells was calculated by extrapolating the internalized nanoparticle concentration within a specific volume of cell populations, and it resulted to be 55.6 °C (PBNP), 52.7 °C (PEG:PB), and 53.3 °C (Exo:PB) (Figure S12). From the experiments, we can show that the Exo:PB particles retain strong photothermal effects, but may also reduce local off-target effects as there is a distinct boundary between laser treated and nontreated regions for Exo:PB. (Figure 4C).

### *In Vivo* Subcutaneous Tumor Targeting and Treatment Utilizing Hybrid Particles

**Figure 5:**
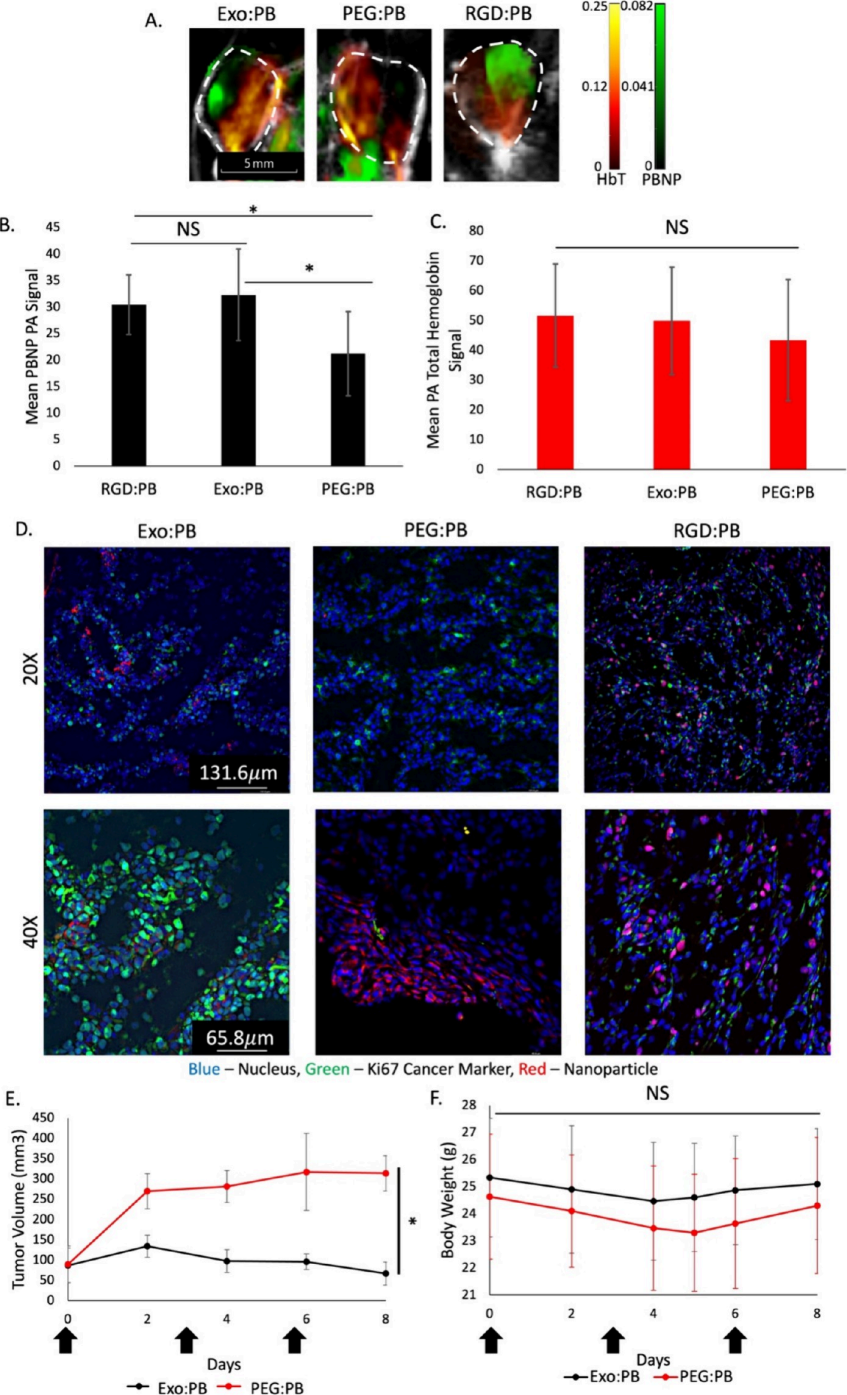
Particle accumulation and therapeutic effect in *in vivo* U-87 subcutaneous model. (A) PA images of subcutaneous U-87 tumors 2 h postinjection with either Exo:PB or PEG:PB particles. Red = total hemoglobin signal. Green = PBNP signal. White dashed line = tumor area. Scale bar is 5 mm. (B) PAI PBNP signal quantification of Exo:PB and PEG:PB in subcutaneous tumors. *N* = 4, *p < 0.05, NS = no significance. (C) PAI total hemoglobin (HbT) signal quantification in subcutaneous tumors. *N* = 4, *p < 0.05, NS = no significance. (D) Immunofluorescence images of subcutaneous tumors 24 h postinjection of DiI-Exo:PB, RITC-PEG:PB, and RITC-RGD:PB taken at 20× and 40×. Red = DiI-Exo:PB, RITC-PEG:PB, or RITC-RGD:PB. Green = Ki67. Blue = cell nucleus. Scale bar = 131.6 μm for 20× images and 65.8 μm for 40× images. (E) Tumor volume in mice during the photothermal treatment period. Black arrows indicate days in which both intravenous injection of Exo:PB (black) or PEG:PB (red) and laser treatment occurred. *N* = 3, *p < 0.01. (F) Average body weight change of mice from Exo:PB (black) and PEG:PB (red) groups. Black arrows indicate days in which mice were intravenously injected with particle and treated with laser.

To first validate the potential of the Exo:PB particles to systemically target glioblastoma tumor cells with distant tissue targeting, a subcutaneous U-87 tumor mouse model was used. The particles were compared to PEG:PB and RGD:PB accumulation patterns to determine overall efficacy. As RGD peptide is a common active targeting moiety for glioblastoma, it is a good control to compare to targeting ability of Exo:PB and their potential use as an alternative for the clinic. We could see PAI signal from PBNPs within the tumor regions starting at 2 h post intravenous (IV) injections for the Exo:PB and RGD:PB particles. In comparison, the signal for IV injected PEG:PB particles were seen mostly on the outer regions of the tumor at the same time point (Figure 5A). With further PAI signal quantification of PBNPs, it is seen that there is statistically significantly more Exo:PB and RGD:PB particle that makes it into the tumor site than PEG:PB at 2 h (Figure 5B). In contrast, the total hemoglobin signal is comparable for the Exo:PB, PEG:PB, and RGD:PB treated mice (Figure 5C), as there was no statistical difference between the three groups. Immune evasion of the exosome from the Exo:PB would allow a longer residence time of the particles in circulation, which led to reaching the target tumor tissues in a higher concentration than the PEG control group as well as similar accumulation to that of RGD:PB. These results are further validated using immunofluorescence staining with Ki67 of the excised tumor tissue. It is seen that there are high concentrations of Exo:PB and RGD:PB present within the tumor. The PEG:PB signal is reduced as most of the signal is seen outside the tumor (Figure 5D). To evaluate the *in vivo* toxicity of the nanoparticles, the liver, brain, heart, lungs, spleen, kidneys, and muscle were excised 24 h after intravenous injection of Exo:PB, PEG:PB, or PBS. Tissue sections were stained using hematoxylin and eosin (H&E) and compared to assess any damage to organs. As seen in Figure S13, there were no changes in tissue morphology for mice injected with Exo:PB or PEG:PB vs the PBS control.

Figure 5E shows the therapeutic potential of Exo:PB particles following intravenous injection and exposure to a 2 W/cm^2^ 808 nm laser for 10 min. During the entire treatment period, body weight was monitored to make sure the mice were healthy (Figure 5F). Treatment started when the tumor size was between 50 and 130 mm^3^ for both Exo:PB and PEG:PB treated groups. After 6 days, it was seen that the Exo:PB treated tumors had returned to baseline tumor size with an average size of ∼80 mm^3^. The PEG:PB group showed less efficient tumor inhibition as tumor size maintained steady growth. As the tumors were exposed to the laser 3 h after injection, this is likely due to the better targeting/accumulation effects of the Exo:PB particles (Figure 5A). Now, to ensure the safety of the treatment due to laser, a separate set of mice were injected with PBS or Exo:PB intratumorally and monitored over 11 days. It is seen that the Exo:PB group shows complete eradication of the tumor mass after treatment, but the PBS control group showed an exponential increase in tumor size (Figure S14). Based on the results, the laser does not cause any skin irritation/necrosis. Apoptotic *ex vivo* analysis of the tumor tissue after intertumoral PTT shows a direct overlay of DiI-Exo:PB with cleaved caspase-3, indicating the particle is the direct cause of tumor cell death (Figure S15).^53^ Therapeutic dose of laser and particle was chosen based on results from Figure S16. There is no statistical difference regarding particle doses (1 to 16 mg/mL) in photoconversion patterns over a 10 min exposure time to a 2 W/cm^2^ laser (Figure S16A). Cell viability is also shown to have no statistical difference when exposed up to 5 W/cm^2^ laser power (Figure S16B). Since we were able to see a therapeutic effect at a lower laser intensity (2 W/cm^2^), it was unnecessary to increase. While the calculated maximum permissible exposure (MPE) for an 808 nm laser is 0.33 W/cm^2^, we chose 2 W/cm^2^ as it is consistent with others in the literature at this stage.^54,55^ For transition to an LITT system, the laser power will be decreased and the time allotted for treatment will be increased to reach the same tumor reduction results.

### Orthotopic Brain Tumor Mouse Model Development and Targeting

**Figure 6:**
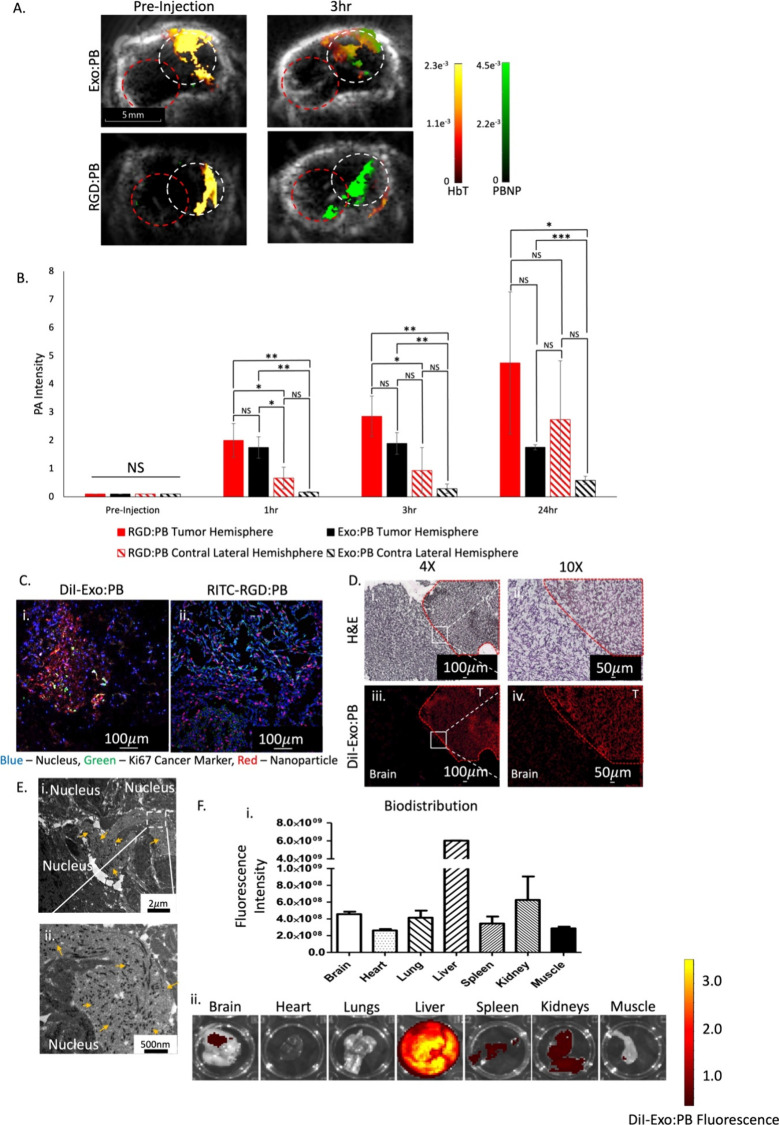
Targeting and biodistribution of Exo:PB in *in vivo* orthotopic brain tumor model. (A) PA images of U-87 orthotopic brain after intravenous injection of Exo:PB and RGD:PB to determine particle accumulation within the brain tumor region. Red = total hemoglobin concentration. Green = PBNP. White circle = tumor hemisphere. Red circle = contra lateral hemisphere. Scale bar = 5 mm. (B) PAI PBNP signal quantification in the tumor hemisphere vs contra lateral hemisphere measured before, 1, 3, and 24 h after intravenous injection. Red = RGD:PB tumor hemisphere. Lined red = RGD:PB contra lateral hemisphere. Black = Exo:PB tumor hemisphere. Lined black = Exo:PB contra lateral hemisphere. *N* = 3, *p < 0.05, **p < 0.01, ***p < 0.001. (C) Immunofluorescence images of (i) Exo:PB and (ii) RGD:PB overlaid with Ki67 24 h postinjection. Red = DiI-Exo:PB or RITC-PEG:PB. Blue = cell nucleus. Green = Ki67. Scale bar = 100 μm. (D) *Ex vivo* brightfield and fluorescence identification of particle accumulation within brain regions. H&E brain tissue brightfield images taken at 4× (i) and 10× (ii). Overlay DiI-Exo:PB (red) fluorescence signal at 4× (iii) and 10× (iv). T = tumor. Scale bar = 100 μm (4×) and 50 μm (10×). (E) BioTEM images taken at (i) 4000× and (ii) 12 000× from the brain tumor region. Yellow arrows indicate PBNPs. Scale bar = 2 μm (4000×) and 500 nm (12 000×). (F) Biodistribution of DiI-Exo:PB particles 24 h after intravenous injection. (i) Quantitative results calculated from fluorescence intensities of extracted organs and (ii) representative *ex vivo* fluorescence images of brain, heart, lungs, liver, spleen, kidneys, and leg muscle. *N* = 3.

Using a previously established protocol, we created an orthotopic glioblastoma model by stereotaxic injection of luciferase-expressing U-87 cell suspensions into the right hemisphere of the mouse brain.^56^ Approximately 2 weeks postoperation, we can start to see a strong luciferase signal within the brain region using an IVIS imaging system. Approximately 3 weeks post inducement, DiI-Exo:PB, RITC-PEG:PB, and RITC-RGD:PB particles were intravenously injected to monitor particle accumulation within the brain tumor region. As seen in Figure 6A, we can see a strong PBNP signal for both Exo:PB and RGD:PB within the brain region 3 h postinjection through PA imaging. This PBNP signal greatly overlays with total hemoglobin (Hb+HbO_2_) blood signals within the brain, which indicates the facilitated intracerebral delivery of the particles through systemic circulation. Further PAI signal quantification of PBNPs was done pre, 1, 3, and 24 h postinjection to quantify the accumulation of Exo:PB and RGD:PB in the brain tumor hemisphere vs the contra lateral brain hemisphere. As RGD peptide is a common targeting agent for glioblastoma due to its ability to pass through the BBB based on many overexpressed integrins such as α_v_β_1_, α_v_β_3_, α_v_β_5_, α_v_β_8_, and α_8_β_1_ within glioblastoma cells.^57,58^ With many RGD particle formulations within advanced FDA-phase trials, the comparison to Exo:PB is valuable to determine the ability to translate into the clinic. At 1 and 3 h postinjection, both Exo:PB and RGD:PB showed statistical differences in accumulation between the tumor and contra lateral hemispheres, but interestingly showed no difference in the tumor hemisphere when compared against each other. At 24 h, the PAI signal for RGD:PB present within the contra lateral hemisphere is no longer statistically different from the tumor hemisphere, indicating that the RGD peptide has off-target effects within the brain. Comparatively, Exo:PB has preferential accumulation within the tumor hemisphere (Figure 6B). As many RGD-recognizing integrins are expressed on many normal cells, such as astrocytes, there is a percentage of RGD peptide that will off-target healthy tissue within the brain which provides inaccurate information for clinicians to identify tumor areas and can cause detrimental amplified photothermal effects.^58^ Further validation of Exo:PB accumulation in the brain was done using inductively coupled plasma mass spectrometry (ICP-MS) at 24 h postinjection (Figure S17), and the results are comparable to that of PAI quantification.

*Ex vivo* analysis shows a nice overlap between DiI-Exo:PB signal and cancer proliferative Ki67 marker, which indicates that we have specific targeting of the Exo:PB particle in the tumor region within the brain (Figure 6Ci). In comparison, RITC-RGD:PB shows off targeting of the U-87 cells as the signal is consistent in tumor and nontumor regions based on overlay with Ki67 (Figure 6Cii). Further H&E staining of brain tissue was done to demarcate tumor and nontumor regions, and DiI fluorescence signal was checked (Figure 6Di and Figure 6Dii). There is a direct overlay with Exo:PB signal and tumor, which can be seen at the borderline of the tumor site (Figure 6Diii and Figure 6Div). At lower magnification, we can see more clearly regions of tumor infiltration with Exo:PB particles with specific and sensitive targeting of glioblastoma cells *in vivo* (Figure S18). Whole-body biodistribution 24 h after injection show Exo:PB particles primarily end up within the liver, but have substantial accumulation (∼4%) within the brain region as well (Figure 6F). Traditional FDA-approved drugs for glioblastoma have low delivery concentrations of <1%.^59^ Therefore, an accumulation of ∼4% within the brain is high in comparison. Now, based on uptake patterns of Exo:PB particles, it is possible that if distribution patterns were evaluated at an earlier time point, the accumulation would be even higher. Liver toxicity may be a concern because it has been shown that PBNPs could cause certain acute liver damage. However, the liver can easily recover from particle exposure as serum indexes of liver functions gradually decrease to normal levels. Thus, intravenous administration of the particles would have minimal effects.^60^ Benefiting from the exosomal transport bypassing BBB while reducing reticuloendothelial system (RES) clearance, there is strong particle signal present in the glioblastoma tumor region. As fluorescence only gives evidence of DiI stained exosomes, not with the particle core, BioTEM was used to cross-validate Exo:PB accumulation. It can easily be seen in Figure 6E that there are cube-shaped PBNP present within the perinuclear area of the tumor cells within the brain. In comparison, PEG:PB showed reduced accumulation in the brain (Figure S19A). It was a concern that the surgery to inoculate tumor cells would cause damage to the BBB and allow for a passive accumulation of particles. Based on *ex vivo* evidence with limited PEG:PB transportation (Figure S19B,C), we can speculate that the BBB was given enough time to heal before experimentation.

Not only do PBNPs have great PA image contrast, they also have MR imaging capabilities. With further doping with Gd^3+^ or Mn^2+^, MRI contrast from the PBNP particle could be increased, and this tool with sensitive soft tissue contrast could be used as a dual-imaging agent (MRI/PAI) that would help validate exact tumor location as well as early detection of cancer.^61^ Simultaneously, metal doping would also allow for the peak light absorbance of particles to be red-shifted (Figure S20) and augment efficacy for FDA-approved, MR-guided laser interstitial thermal therapy (e.g., NeuroBlate, Visualase), which functions at wavelengths 800 nm through 1064 nm.^62^ While local inflammation can be a common side-effect of these types of ablation therapies, PBNPs can help mitigate this as they have shown to have enzymatic activity based on the alternating Fe^2+^/Fe^3+^ surface valences (Figure S21) that can have both anti-inflammatory effects (Figure S22) and reduce tumor hypoxia (Figure S23).^10^ Multispectral PA imaging can be further applied to real-time track tumor hypoxia associated with tumor progression and treatment monitoring (Figure S23).^63^

The Exo:PB particles discussed in this paper have various future applications. For example, the hybrid particles could improve the efficacy and safety of MR-guided LITT in the brain.^16,26^ By changing the cell type from which the exosomes are derived, the hybrid nanoparticles can achieve targeting of different cancers.^64,65^ With the specificity of targeting, these particles could be used during surgical resection to help identify the tumor borders and areas of infiltration.^66^ Further modification of the PBNPs could be done to make hollow interior and load hydrophobic chemotherapeutics (e.g., temozolomide) and allow for a targeting drug delivery to bypass the BBB.^13^ This would be a valuable combination between PTT and chemotherapy to combat the aggressiveness of this devastating cancer.

## Conclusions

We have developed an Exo:PB nanohybrid that benefits from the innate properties of both PBNPs and U-87 derived exosomes. The Exo:PB particles can easily be manufactured with a full exosome coating, as seen through DLS, NTA, TEM, electron mapping, and Western blot. When these particles are exposed to an 808 nm laser, we can see localized cell death in both *in vitro* and *in vivo* experimental setups. Once introduced into an orthotopic glioblastoma mouse model, we can see selective targeting of nanohybrids to brain tumors with increased contrast using PAI. With all the components of the particles constructed by biologically driven materials (exosome) or FDA-approved biocompatible agents (PBNPs), this material is potentially applicable in the clinic. Overall, the Exo:PB nanohybrids we present here can be used as a GBM-specific theranostic agent that can enhance noninvasive diagnostics by PAI and act as simultaneous photothermal ablation agents.

## Materials and Methods

### Materials

Iron chloride (catalog no. 236489), potassium hexacyanoferrate(II) trihydrate (catalog no. P9387), 1,1′-dioctadecyl-3,3,3′,3′-tetramethylindocarbocyanine perchlorate (DiI, catalog no. 468495), fetal bovine serum (FBS, catalog no. SH30396.03), penicillin–streptomycin (catalog no. 15140-122), citric acid (catalog no. 251275), polyvinylpyrrolidone (MW ∼ 40 000, catalog no. PVP40), polybis(amine) MW 2000 (catalog no. 14501), phosphate buffered saline (PBS, catalog no. D8537), 3-(4,5-dimethylthiazolyl-2)-25-diphenyl tetrazolium bromide (MTT, catalog no. 102227), dimethyl sulfoxide (DMSO, catalog no. 276855), acetone (catalog no. 270725), 30% hydrogen peroxide (catalog no. 216763), Matrigel (catalog no. 354234), hematoxylin (catalog no. 65065-M), eosin Y solution (catalog no. 586X), *N*-scetyl-l-cysteine (NAC, catalog no. A9165), calcein AM (catalog no. 206700), dodecyltrimethylammounium bromide (CTAB, catalog no. D8638), sodium borohydride (catalog no. JS-S2490), gold(III) chloride trihydrate (catalog no. 520918), l-ascorbic acid (catalog no. A7506), silver nitrate (catalog no. 209139), rhodamine B isothiocyanate (catalog no. 283924), diethyl ether (catalog no. 309966), ethyl acetate (catalog no. 319902), methanol (HPLC grade) (catalog no. A452SK), gadolinium(III) chloride (catalog no. G7532), potassium hexacyanoferrate (III) (catalog no. P8131), hematoxylin solution (catalog no. 51275), and eosin Y (catalog no. HT110216) were purchased from Sigma-Aldrich Chemicals (St. Louis, MO). Eagle’s minimal essential medium (EMEM, catalog no. MT10009CV), paraformaldehyde (catalog no. 19202), glutaraldehyde (catalog no. O2957-1), propidium iodide (catalog no. J66584), Invitrogen NucBlue Live ReadyProbes reagent (Hoechst, 33342, catalog no. R37605), Invitrogen CM-H2DCFDA (catalog no. C6827), caspase-3 antibody (catalog no. NC1215364), anti-Ki67 antibody (catalog no. MA5-14520), lipopolysaccharide (LPS, catalog no. 00-497693), triethylamine (catalog no. T0886), and hydrochloric acid (catalog no. HX0603) were purchased from Thermo-Fisher Scientific (Waltham, MA). 2% uranyl acetate (catalog no. 102092-284) was purchased from VWR (Radnor, PA). Catalase assay kit (catalog no. 707002) was purchased from Cayman Chemical Company (Ann Arbor, MI). OxiVision Green hydrogen peroxide sensor (catalog no. 21505) was purchased from AAT Bioquest (Pleasanton, CA). Nuclear Fast Red, 1% solution (catalog no. 24199C-250) was purchased from Polysciences (Warrington, PA). RGD peptide (catalog no. 350362) was purchased from ABBIOTEC. U-87 MG (catalog no. HBT-14) and RAW 264.7 (catalog no. TIB-71) cells lines were purchased from ATCC. All chemicals were of high purity, and all dilutions were done using DDI water.

### Prussian Blue Nanoparticle Synthesis

Prussian Blue nanoparticle (PBNP) synthesis was performed using a co-precipitation reaction, where iron chloride (1 mM) is mixed with potassium hexacyanoferrate(II) trihydrate (1 mM) in the presence of citric acid. The solution is then left stirring at 60 °C overnight. The next day, the PBNP reaction mixture is washed with water and equal part of acetone at 12 000 rpm for 20 min (×3). The final PBNP solution is suspended in water.

### PEGylated Prussian Blue Nanoparticle Preparation

PEGylated Prussian Blue nanoparticles (PEG:PB) were prepared via a two-step synthesis with some modifications.^67^ Initially, PBNPs were synthesized to include a PVP coating using a coprecipitation reaction. Iron(III) chloride (1 mM) is mixed with potassium hexacyanoferrate (II) trihydrate (1 mM) while in the presence of PVP and stirred at 60 °C overnight. The next day, the reaction mixture was washed with a 1:1 water and acetone mixture at 12,000 rpm for 20 min (×3). Particles were then PEGylated by doing a surfactant substitution. PVP–PBNP (2 mg/mL) was mixed with an equal amount of poly bis(amine) and stirred at RT for 24 h. The next day, the PEG:PB particles were washed with DDI water (×3). Final solution was suspended in water. Conjugation was validated using a PerkinElmer Spectrum 2 FTIR through a drop cast method.

### Conjugation of RITC-RGD

To a single vial, 2 mg of RGD peptide, DMSO, and triethylamine was mixed until the RGD peptide was fully dissolved. In a separate vial, 3 mg of RITC and DMSO were mixed. The two solutions are then combined and mixed at RT for 24 h. The next day, the conjugate was purified using a diethyl ether precipitation process and a rotovap was used to removed excess DMSO in the presence of methanol. The molecular weight of the sample was verified using a Waters G2-XS-Q-ToF mass spectrometer with a Waters Acquity UPLC (flow rate: 0.2 mL/min in 1:9 water:methanol + 0.1% formic acid). Calculated weight for C_41_H_52_,N_9_O_9_S (M + H^+^): 846.3603. Mass spectrometry weight: 846.3606.

### RITC Conjugated RGD Peptide Prussian Blue Nanoparticle Preparation

RGD peptide and PBNPs were combined in a 1:200 (peptide:nanoparticle) volumetric ratio in 4 mM borate buffer as previously reported with a similar method.^68^ The solution was left to mix overnight at RT. The following day, the particle mixture was washed at 12 000 rpm for 30 min (×2) with DDI water. Final RITC-RGD:PB particles are stored in water. Conjugation was validated using a PerkinElmer Spectrum 2 FTIR through a drop cast method.

### Isolation of U-87 Exosomes

Initially, U-87 MG cells were plated in 100 cm^2^ dishes in EMEM medium supplemented with 10% fetal bovine serum. After incubation for 1 day at 37 °C and 5% humidity, the cells were washed, and the medium was replaced with EV-depleted EMEM medium. After another day, the medium was taken and placed in a 50 mL conical tube and centrifuged for 10 min at 600*g* to remove any cells. The supernatant was then centrifuged at 2000*g* for 30 min to remove apoptotic bodies, 20 000*g* for 60 min to remove microvesicles, and finally at 100 000*g* for 60 min to isolate the U-87 exosomes in the form of a pellet. Exosomes are stored in PBS at −80 °C until used.

### Exosome-Coated Prussian Blue Nanoparticle Preparation

Exosome-coated Prussian Blue nanoparticles (Exo:PB) were prepared through physical extrusion. Initially, 1.5 mg/mL citric capped PBNPs are mixed with 1 mL of U-87 derived exosomes (1 × 10^9^ particles/mL). The particle mixture is then extruded using an Avanti Polar Lipids Mini Extruder (catalog no. 610023) using a 200 nm PC membrane (catalog no. 610006) for 11 passages at room temperature (Figure S5). Following extrusion, the suspension is washed at 12 000 rpm for 20 min to remove any unused exosomes and resuspended in 1 mL water. For fluorescent labeling, 1 mg/mL 1,1′-dioctadecyl-3,3,3′,3′-tetramethylindocarbocyanine perchlorate (DiI) is added and incubated at 37 °C for 1 h. The particles are then washed ×2 with water at 12 000 rpm for 20 min. Final Exo:PB and DiI-Exo:PB solutions are suspended in water.

### Characterization of U-87 Exosomes, PBNP, PEG:PB, Exo:PB, RGD:PB, and Gd:PB Particles

Using Nanoparticle Tracking Analyzer (NTA, ZetaView), the size and quantity of U-87 derived exosomes was determined. Dynamic light scattering (DLS, Zeta Sizer Nano, Malvern Instruments) was used to determined hydrodynamic size and ζ potential values for all particles. Size, morphology, dispersity, and composition were determined using a 2200FS transmission electron microscopy (TEM, JEOL) with energy-dispersive X-ray spectroscopy (EDX) capabilities. Samples for electron mapping were prepared using a uranyl acetate staining method. Initially, Exo:PB particles were mixed with equal volume 2% PFA and added to a 300 mesh copper grid. The grid is left to dry for 20 min in a fume hood and then washed with PBS. 1% glutaraldehyde is added to the grid and left to dry for 5 min. Following fixation of the particles, the grid is washed ×8 with DDI water. Finally, 2% uranyl acetate is added to the grid and left to sit for 1 min. All steps for the uranyl acetate staining protocol were performed in a fume hood.

### Gold Nanorod Synthesis

Using a seed-mediated growth method, gold nanorods (AuNRs) were synthesized using a previous established method with some modifications.^69^ Initially, a seed solution containing 2.5 mL of gold(III) chloride (0.1 mM), 5 mL of CTAB (2 mM), 600 μL of sodium borohydride (10 mM), and 2.5 mL of water is prepared. Next, a separate growth solution is prepared where 460 μL of silver nitrate (100 mM), 5.1 mL of ascorbic acid (87 mM), and 1.8 mL of seed solution are added to 740 mL of CTAB (2 mM). The solution is left overnight to react. The next day, the solution is washed ×3 with water at 10 000 rpm for 10 min. The final solution is stored in water.

### Particle Based Photothermal Capabilities

1 mL of PBNPs (0, 1, 2, 4, 8, and 16 mg/mL) was exposed to an 808 nm laser at 2 W/cm^2^ for 10 min to determine overall changes of temperature. Samples were placed 1 in. from the output of the laser, and the rate of temperature change was monitored every 1 min using a hand-held Cx series FLIR thermal camera. Photothermal stability of AuNR and PBNP was determined by exposing 1 mL (0.5 mg/mL) solutions to an 808 nm laser at 2 W/cm^2^ for 10 min increments, where every increment was followed by a 5 min cooldown where the laser is turned off. PBNP, PEG:PB, and Exo:PB (1 mg/mL) particles were also exposed to an 808 nm laser for 10 min and left to cool for 10 min where thermal images were taken every minute for 10 min during the heating process. Finally, the size of the particles was examined before and after exposure to the laser. Particles were exposed to the 808 nm laser at 2 W/cm^2^ for 1 min. DLS measurements were taken of the particles before and after exposure.

### Cell Based Maximum Temperature Extrapolation

Initially, U-87 cells were seeded at 30 000 cells/well and left to incubate overnight at 37 °C and 5% humidity. The next day, cells were treated with 1.5 mg/mL of PBNP, PEG:PB, Exo:PB, or nothing and left to incubate for another 24 h at 37 °C and 5% humidity. On the last day, the cells were detached with 0.05% trypsin and centrifuged to obtain a cell pellet. The cell pellets (*n* = 3 for each condition) were then resuspended in 100 μL of EMEM media and exposed to an 808 nm laser (2 W/cm^2^) for 1 min. Temperature of the cell pellets were taken before and after laser exposure. Concentration of particle within the cell pellet was back calculated using a concentration vs change in temperature (Δ*T*) curve for each particle type. With a consistent concentration of 0.1 mg/mL within the cell pellet, a separate volume vs Δ*T* curve was constructed and the maximum temperature generated within U-87 cells (*V* = 4.6875 × 10^–5^ mm^3^ as determined from Figure 2G) was calculated for uptake of PBNP, PEG:PB, or Exo:PB.

### Photothermal Conversion Efficiency Calculation

To determine the photothermal conversion efficiency (η) of PBNP, PEG:PB, Exo:PB, and AuNRs, results from Figure 3A of Figure S4A were analyzed. Considering the first photothermal cycle for each of the particles, the cooling cycle (highlighted in blue) was used to determine θ from eq 1. *T* (°C) = temperature at any time point within the cooling cycle, *T*_surr_ (°C) = temperature of the solvent, *T*_max_ (°C) = maximum temperature reached within the cooling cycle. *T*_surr_ was determined using a vial of water under the same conditions.
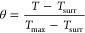
1Once θ was calculated for every given temperature within the cooling cycle, a τ (s) time constant is determined using the inverse relationship between the time and −ln(θ) of the same cycle. τ is found to be the slope of the linear correlation, which is seen in eq 2. *t* (s) = any given time during the cooling period.
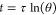
2After deducing the value of τ for each of the particles, the value for *hs* (J/s°C) can be calculated. *hs* is represented by the heat transfer coefficient (*h*) and total surface area of the solution (*s*) and is calculated using eq 3. *m* (g) = mass of the solution, *C* (J/g °C) = specific heat capacity of the solution.
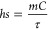
3Finally, the photothermal conversion efficiency can be calculated using eq 4. *Q*_surr_ (J/s) was determined from a vial of water exposed to the same conditions. *I* (W) = laser power, *A*_808_ = absorbance of the particle solution at 808 nm.
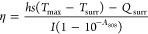
4

### Cell Uptake

U-87 cells were seeded in a 4-chamber slide at 10 000 cells/well and left overnight in a cell incubator at 37 °C and 5% humidity. The next day, cells were treated with 0.1 mg/mL of DiI U-87 exosomes, DiI-Exo:PB, or RITC conjugated PEG:PB and then left for another 24 h at 37 °C and 5% humidity. On the third day, the cells were washed and stained with 1 μM calcein AM and mounted using Prolong Gold reagent with DAPI. Images were taken using THUNDER microscopy (Leica Microscopy).

### *In Vitro* BBB Model

Initially, U-87 cells were plated in 24-well plates with 8 μm well inserts containing either a polycarbonate or basement membrane at 20 000 cells/well. The cells were then left to incubate overnight at 37 °C and 5% humidity. The next day, DiI-Exo:PB, RITC-PEG:PB, and RITC-RGD:PB (0.1 mg/mL) were added into the well insert. On the third day, the well inserts were removed, and cells were stained with DAPI and calcein AM. Images were obtained using Keyence microscopy.

### Time-Based Cell Uptake

U-87 cells were seeded in a 96-well plate at 10 000 cells/well and left overnight at 37 °C and 5% humidity. The following day, wells were treated with DiI-Exo:PB (1.5 mg/mL). At each time point of 1, 2, 3, 4, 5, 6, 7, 8, 9, 10, 11, 12, 24, and 48 h, the cells were washed with PBS and the particle fluorescence was obtained and compared to a standard curve.

### Cellular Tumorigenesis

Initially, U-87 cells were seeded in a 96-well plate at 10 000 cells/well and left in a cell incubator overnight at 37 °C and 5% humidity. The following day, cells were treated with either Exo:PB or PEG:PB (0.1 mg/mL). Finally, 24 and 48 h after treatment, the cells are washed with PBS and stained with calcein AM. Fluorescence intensity of the calcein AM was taken and compared to cells that have not been treated.

### Cell Viability

Initially, cells were seeded in a 96-well plate at 20 000 cells/well and left in a cell incubator overnight at 37 °C and 5% humidity. The next day, cells were treated with 0, 0.031 25, 0.0625, 0.125, or 0.25 mg/mL of U-87 exosomes, PBNPs, PEG:PB, or Exo:PB and then left for another 24 h at 37 °C and 5% humidity. On the last day, the supernatant is discarded and replaced with an MTT solution and left to incubate for 4 h. The MTT solution was then removed, and the formed formazan crystals were dissolved using DMSO and absorbance was measured at 570 nm using a SoftMax Pro plate reader (Molecular Devices, CA). For cells that received both particle and laser treatment, on the last day cells were exposed to an 808 nm laser at 1.5 W/cm^2^ for 1 min and then the MTT assay was performed.

### Live and Dead Cell Assay

U-87 cells were seeded in a 24-well plate at 30 000 cells/well and left overnight in a cell incubator at 37 °C and 5% humidity. The following day, cells were treated with 1 mg/mL of particle and incubated for 1 h. The cells were then washed with PBS then treated with an 808 nm laser at 2 W/cm^2^ for 1 min and left to incubate for overnight at 37 °C and 5% humidity. On the third day, the cells were washed and stained with 1 μM calcein AM and 2 μM propidium iodide and imaged using Keyence fluorescence microscopy.

### Catalase Activity

Using a Cayman Chemical catalase assay kit, the catalase activity of our PBNP and Exo:PB particles was measured at different concentrations and compared to a catalase control. All samples are read off a newly prepared standard curve.

### OxiVision Peroxide Assay

PBNP and Exo:PB particles at 1 mg/mL were added to a solution of 5 μM OxiVision Green dye and 10 mM hydrogen peroxide and left for 20 min at room temperature. Fluorescent intensity values were obtained using an excitation of 490 nm and emission of 525 nm.

### DCFDA Assay

Raw 264.7 cells were seeded at 30 000 cells/well in a 24-well plate and left overnight in a cell incubator at 37 °C and 5% humidity. The next day, the cells were treated with 1 μg/mL of LPS and 1 mg/mL of either PBNP or Exo:PB then left to incubate at 37 °C and 5% humidity for another 24 h. On the final day, the cells were washed and treated with DCFDA for 30 min. For the LPS + NAC group, 30 mM NAC was treated for 30 min prior. Fluorescent intensity was measured at an excitation of 485 nm and emission of 535 nm. Images were taken using Keyence microscopy.

### *In Vivo* Subcutaneous Tumor Model

All animal studies performed were approved by the Institutional Animal Care and Use Committee (IACUC) at Michigan State University, while animal care and wellbeing throughout the study was monitored by the Center for Animal Resources (CAR) at Michigan State University. Nude male mice were purchased from The Jackson Laboratory. Animal experiments were done with at least an *N* = 3. Before subcutaneous tumor implantation, luciferase expressing U-87 cells (500 000 cells/tumor) using the Sleeping Beauty transposon were mixed with Matrigel in a 1:1 volumetric ratio.^70^ Cells were then injected into the flank region of the mouse. Tumors were visible after ∼1 week post inducement. All mice were anesthetized using an isoflurane/oxygen mixture during all procedures.

### Intertumoral Injection *In Vivo* Photothermal Treatment

1 mg/mL (100 μL) Exo:PB, PEG:PB, or PBS was injected intratumorally on day 0 and day 7 and then treated with an 808 nm laser at 2 W/cm^2^ for 1 min. Tumor size was measured every day for 13 days using a sliding vernier caliper and compared with luciferase signals using IVIS imaging (PerkinElmer Inc., Waltham, MA).

### Intravenous Injection *In Vivo* Photothermal Treatment

10 mg/mL (100 uL) of Exo:PB, PEG:PB, or RGD:PB was injected intravenously on days 0, 3, and 6. Three hours after injection, tumors were exposed to an 808 nm laser at 2 W/cm^2^ for 10 min. Tumor size and body weight were measured every 2 days with a vernier caliper and standard open benchtop scale. On day 6, tumor size and body weight were determined before treatment.

### *In Vivo* Toxicity

10 mg/mL (100 uL) of Exo:PB, PEG:PB, or RGD:PB was injected intravenously and 24 h later the mice were sacrificed. The liver, brain, heart, lungs, spleen, kidneys, and leg muscle were excised and sectioned at 6 μm thickness. Tissue sections were then stained with hematoxylin and eosin (H&E) solutions with staining times of between 1 and 10 s for hematoxylin and 30 s for eosin.

### MSOT Imaging

Photoacoustic imaging was performed using inVision 512-echo preclinical multispectral optoacoustic tomographic imaging (MSOT) system (iThera Medical, Germany). Mice were submerged in a water tank in a horizontal position in a holder and were wrapped in a thin polyethylene membrane to prohibit direct contact between water and mouse. Anesthesia (2% isoflurane) and oxygen are supplied through a breathing mask. The images were taken preinjection, 0, 1, 2, 3, 6, 12, 24 h after injection of 10 mg/mL (100 μL) of nanoparticles in mice. Imaging was performed 0.2 mm steps, and all acquisition was performed using 10 averages per illumination wavelength, with the wavelengths chosen as follows: 680, 700, 730, 760, 800, and 850 nm. This resulted in an acquisition time of less than 10 min. Image analysis was performed by using ViewMSOT software.

### *In Vivo* Orthotopic Brain Tumor Model

Initially, the mouse would be anesthetized using isoflurane at 2 L/min. Meloxicam is then administered through intraperitoneal injection at a concentration 1 mg per mouse. The mouse is transferred to a stereotaxic device and an incision is made above the top side of the skull. A 10 μL needle is adjusted to 2 mm *x*, 1.5 mm *y* from the bregma and the skull is punctured using a small gauge needle. The needle is then lowered into the hole to 2.5 mm, then 3 μL of 3 × 10^5^ luciferase expressing U-87 cells is injected at a rate of 0.5 μL/min. The needle is left to sit for 5 min postinjection and then pulled out at a rate of 1 mm/min. The mouse is then removed from the stereotaxic device and the incision is stitched together.^56^ Finally, the mouse is left to recover on a bed that is 37 °C. Luciferase signal within the brain region is checked 1–2 weeks postsurgery to determine tumor development.

### *In Vivo* Orthotopic Brain Particle Accumulation

Mice were anesthetized using 2 L/min isoflurane. 2 mg of DiI-Exo:PB, RITC-PEG:PB, or RITC-RGD:PB was injected through iv. PAI images were taken before injection and 3 h after injection.

### *Ex Vivo* Immunohistology

After experimentation, mice were sacrificed and the tumors or brains were removed and sectioned. H&E staining was done by removing the OCT layer from the tissue, staining with hematoxylin for 45 s followed by multiple washes with water, and 30 s of staining with eosin followed by washing multiple times with ethanol and final fixing using xylene glue. Images were taken using Keyence microscopy. Immunofluorescence staining was performed for apoptosis using anticleaved caspase-3 antibody and tumor marker using anti-Ki67 antibody incubated overnight at 4 °C. After washing with PBS, Alexa 488 conjugated secondary antibodies were incubated 1 h at room temperature. After staining, the slides were mounted using Prolong Gold reagent with DAPI. Fluorescence images were taken using THUNDER microscopy (Leica Microsystems, Germany).

### BioTEM Sample Preparation and Imaging

Following experimentation, mice were sacrificed, and the brains were removed and placed in 4% PFA. Following fixation with PFA, a small portion of the brain tumor region was taken and resuspended in a 2.5% glutaraldehyde (0.1 M cacodylate buffer) overnight at room temperature. The next day, samples were washed 3 times with 0.1 M cacodylate buffer for 10 min each. This was followed with postfixation in 1% osmium tetraoxide (0.1 M cacodylate buffer) for 2 h and then washed 3 times with 0.1 M cacodylate buffer for 10 min each. Samples are then dehydrated with acetone from 50 to 100°*C*. Finally, spurr resin is used to infiltrate the samples while decreasing the amount of acetone every 2–3 h and increasing the amount of spurr resin proportionally. The resulting spurr resin blocks are left in an oven for 24 h and then sectioned using an RMC MYX ultramicrotome (Boeckeler Instruments). Images were taken on JEOL 1400 Flash TEM.

### Biodistribution

24 h after injection with Exo:PB particle, the mice were sacrificed and the brain, heart, lungs, kidneys, spleen, liver, and portion of leg muscle were taken. Organs were placed in separate wells of a 24 well plate and fluorescence was measured using IVIS imaging.

### ICP Sample Preparation and Measurements

24 h after injection with Exo:PB particle, the brain was harvested and cut into two: brain tumor hemisphere and contra lateral hemisphere. Using a CEM Mars6 microwave digestion system, the hemispheres were liquified in pure nitric acid. Samples were then diluted and run through an Agilent 8900 QQQ-ICP-MS and compared to a prepared iron standard curve. All samples were weighed before and after each step.

### Synthesis of Gadolinium Doped Prussian Blue Nanoparticles

Gd:PB particles were synthesized using a co-precipitation method between gadolinium(III) chloride and potassium(III) ferricyanide in the presence of citric acid and polyvinylpyrrolidone. A solution of gadolinium(III) chloride, citric acid, and hydrochloric acid is slowly added dropwise to another solution containing potassium(III) ferricyanide, polyvinylpyrrolidone, and hydrochloric acid. The solution is stirred at 2 h at RT and then stirred at 60 °C overnight. The following day, the particles are washed at 12 000 rpm for 40 min (×2) with DDI water. The final Gd:PB particle solution is stored in water.

### Statistical Analysis

Cell based experiments were performed in a sterile environment and done with at least *n* = 6. Statistical analyses were performed using Excel or Graphad Prism software for one-tailed or two-tailed *t* test analysis. *P* values less than 0.05 were considered significant.

## Supporting Information Available

PBNP, Exo:PB, and PEG:PB DLS and corresponding ζ potentials; Exo:PB size stability; Western blot information for U-87 exosomes, Exo:PB, and U-87 cell lysate; U-87 exomsome TEM image; FTIR results for PBNP, PEG:PB, RITC-RGD, and PVP:PB; reaction scheme for RITC-RGD conjugation; mass spectrometry results for RITC-RGD; TEM image, absorbance curve, DLS, and fluorescamine assay results for RITC-RGD:PB; graphical representation of exosome isolation and Exo:PB particle formation; EDX spectrum for uranyl-acetate stained Exo:PB; timed cellular uptake for Exo:PB in U-87 cells; *in vitro* BBB results for RITC-PEG:PB and RITC-RGD:PB; AuNR and PBNP temperature gradient comparison with corresponding AuNR TEM images before and after laser exposure; thermal image profile for PBNP, PEG:PB, and Exo:PB; single cell maximum temperature estimation; H&E-stained tissue from subcutaneous tumor bearing mice; intravenous injection of PBS in U-87 subcutaneous mice and corresponding weight; intratumoral injection of Exo:PB and PEG:PB in subcutaneous tumor bearing mice for photothermal therapy; concentration–temperature profile for PBNPs; U-87 cell viability vs laser intensity; ICP results for Exo:PB in tumor and contra lateral tissue; heavy metal stained brightfield and overlay DiI-Exo:PB fluorescence images; PA images of a mouse brain before PEG:PB intravenous injection and 3 h after injection; PEG:PB brain *ex vivo* analysis with Ki67 marker; BioTEM images of brain after PEG:PB injection; TEM image, DLS, and absorbance curve for Gd:PB; catalase and hydrogen peroxide degradation assay results using PBNP and Exo:PB; DCFDA cell assay results with PBNP and Exo:PB; PA subcutaneous images before and after photothermal treatment with Exo:PB (PDF)
