# Supplementary material for: Exosome-Coated Prussian Blue Nanoparticles for Specific Targeting and Treatment of Glioblastoma
Source: ACS Appl Mater Interfaces. 2024 Apr 10;16(16):20286–301. doi: 10.1021/acsami.4c02364 (PMC11056931; doi:10.1021/acsami.4c02364)
Supplement: Supplementary file 1 — am4c02364_si_001.pdf [file am4c02364_si_001.pdf]

Supporting Information for:

Exosome-Coated Prussian Blue Nanoparticles for Specific Targeting and Treatment of Glioblastoma

Meghan L. Hill[1,4], Seock-Jin Chung[1,4], Hyun-Joo Woo[1,4], Chorong Park[1,4], Kay Hadrick[1,4], Md Nafiujjaman[1,4], Panangattukara Prabhakaran Praveen Kumar[1,4], Leila Mwangi[2,4], Rachna Parikh[3,4], and Taeho Kim[1,4*]

[1]Department of Biomedical Engineering, [2]Department of Chemical Engineering and Materials Science, [3]Department of Human Biology, Lyman Briggs Honors College, [4]Institute for Quantitative Health Science and Engineering, Michigan State University, East Lansing, Michigan 48824

Corresponding author: Taeho Kim (kimtae47@msu.edu)

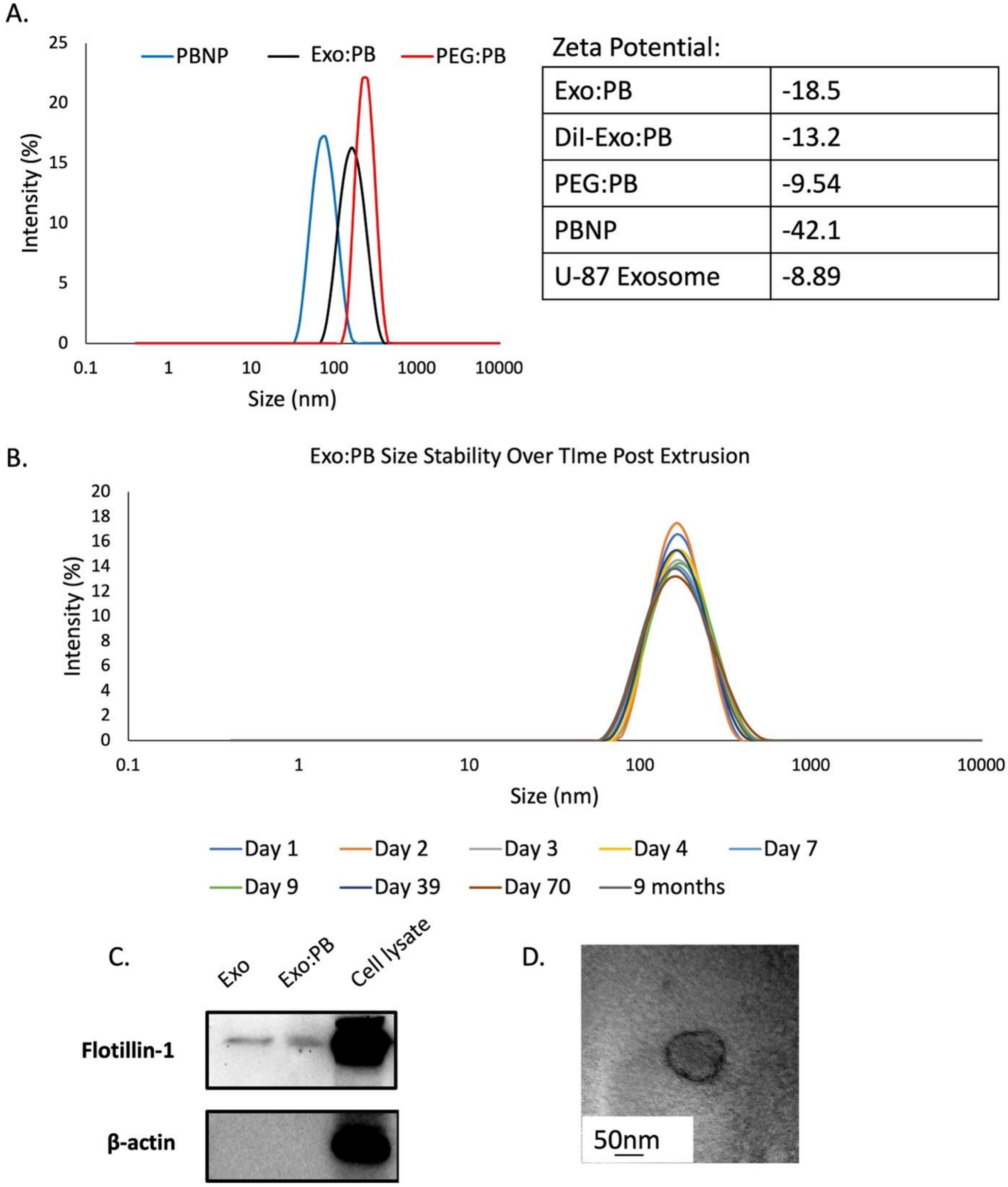

Zeta Potential:

Exo:PB	-18.5
DiI-Exo:PB	-13.2
PEG:PB	-9.54
PBNP	-42.1
U-87 Exosome	-8.89

Figure S1: Particle Characterization. (A) DLS size distribution data of PBNP (blue), Exo:PB (black), and PEG:PB (red) with corresponding zeta potential values. (B) DLS size distribution of Exo:PB after storage at 4 °C for up to nine months. (C) Western blot analysis of U-87 derived exosomes and Exo:PB vs. U-87 cell lysate. Flotillin-1: exosome marker, β-actin: cell membrane marker. (D) TEM image of a uranyl acetate-stained U-87 exosome.

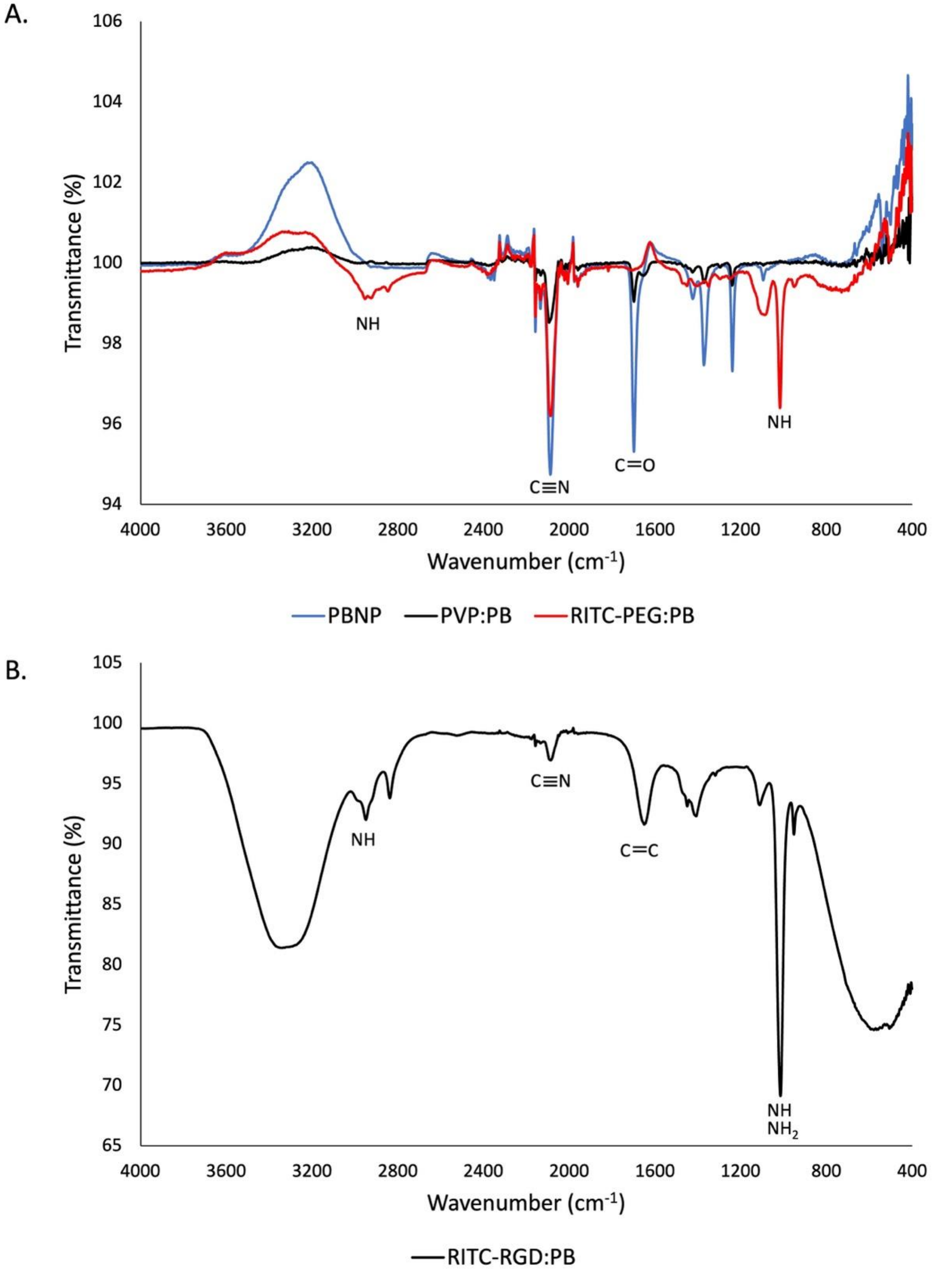

Figure S2: FTIR Particle Characterization. (A) FTIR spectrum of PBNP (blue), PVP:PB (black), and RITC-PEG:PB (red). Primary identification peaks are; NH (2930-2950 cm^{-1} and 1014 cm^{-1}, PEG), CN (2089 cm^{-1}, PBNP), C=O (1699 cm^{-1}, citric acid/PVP). (B) FTIR spectrum of RITC-RGD:PB. Primary identification peaks are: NH(2930-2950 cm^{-1} and 1014 cm^{-1}, RGD), CN (2089 cm^{-1}, PBNP), C=C (1699 cm^{-1}, RITC).

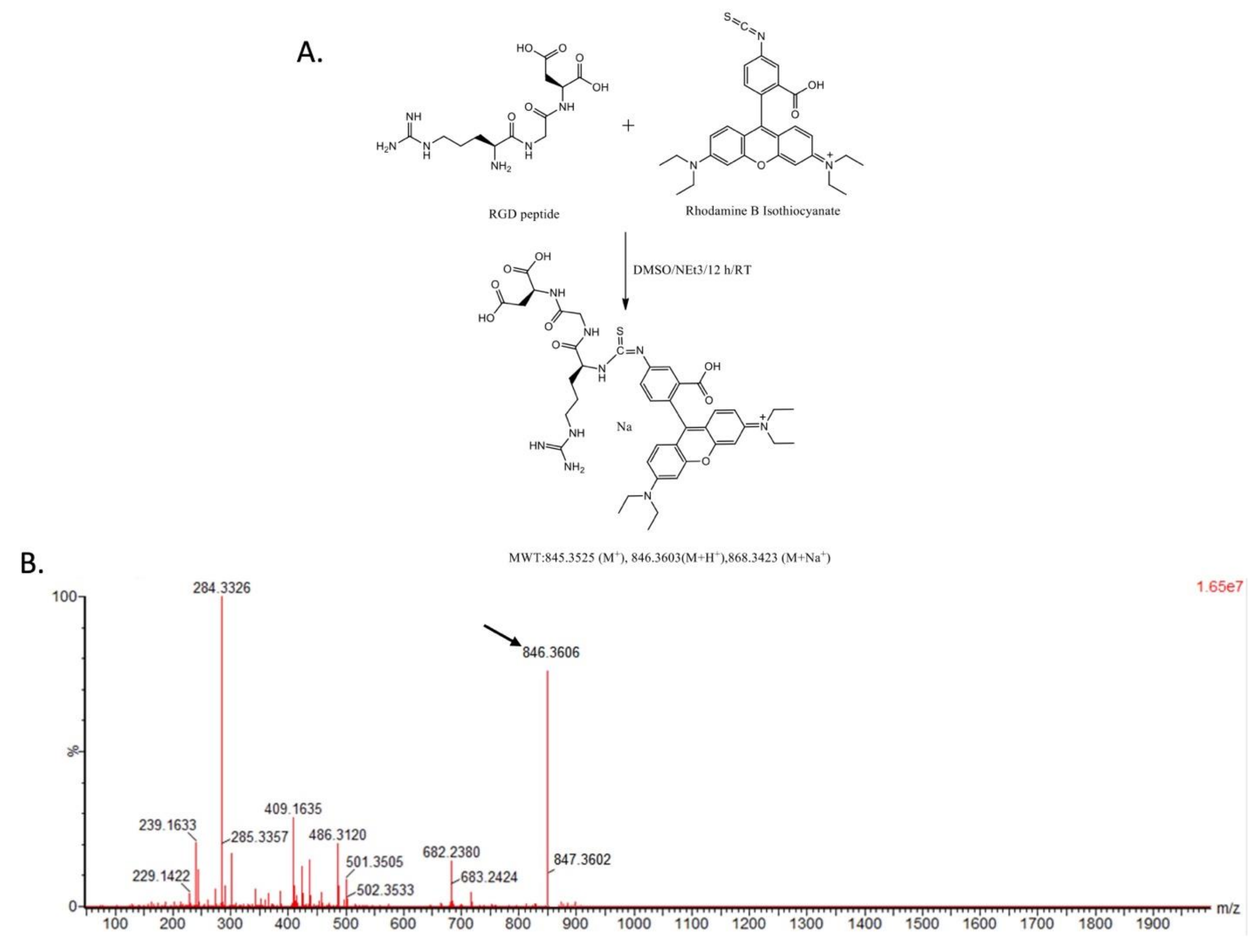

Figure S3: RITC-RGD Conjugate Validation. (A) Reaction scheme between RGD peptide and RITC to create RITC-RGD. (B) Mass spectrometry results for RITC-RGD. Molecular weight (846.3606 g/mol) is indicated with a black arrow.

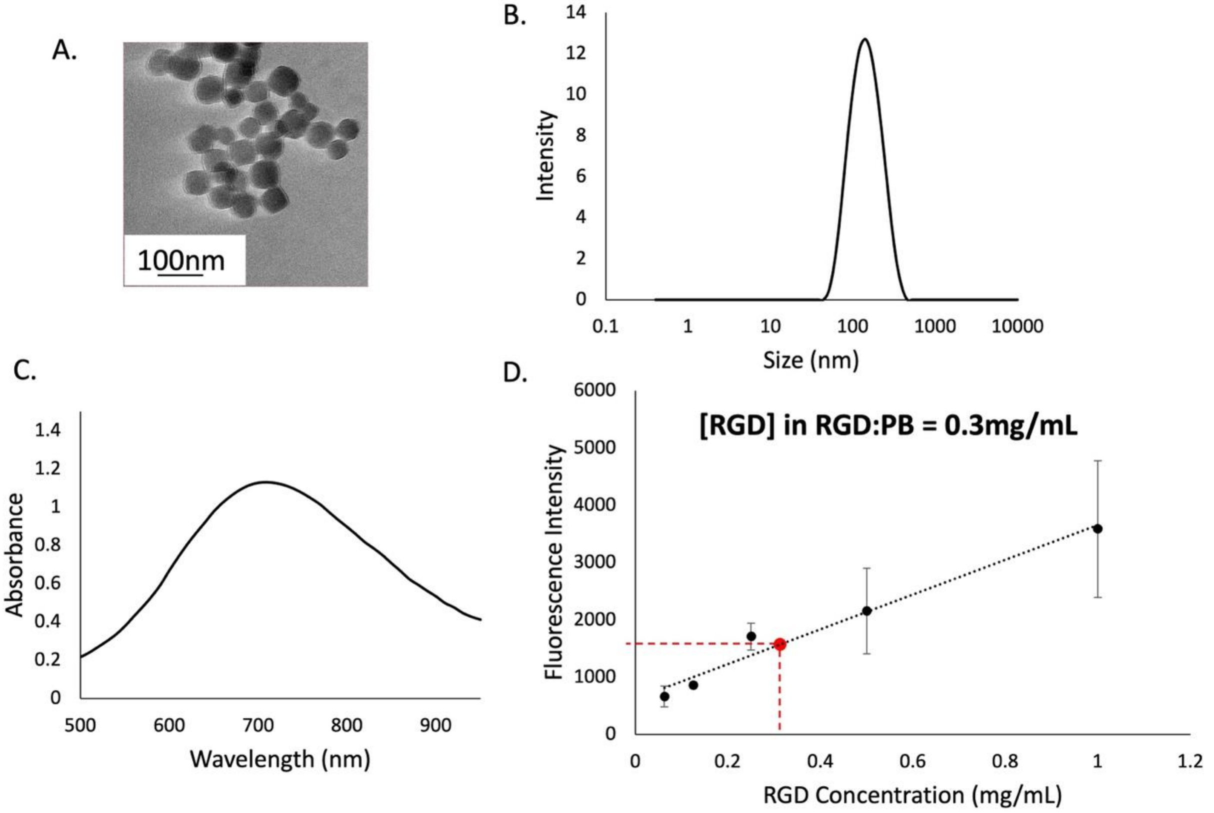

Figure S4: RGD:PB Particle Characterization. (A) TEM images of RGD:PB particles. Scale = 100 nm. (B) DLS size measurement and (C) absorbance curve of RGD:PB particles. (D) Fluorescamine assay results for RGD:PB to quantify the amount of RGD peptide present. Red dot indicates RGD:PB.

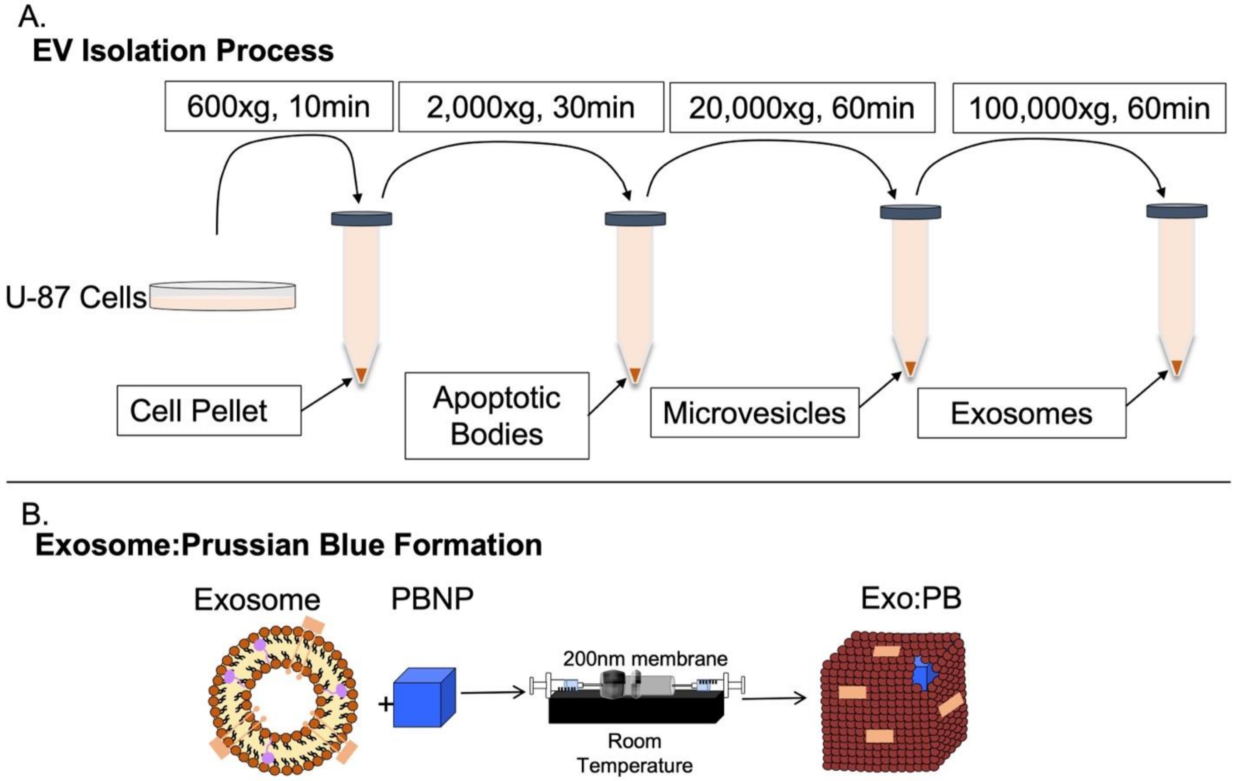

Figure S5: Graphical representation of experimental methods. (A) Exosome isolation procedure and (B) Exo:PB particle formation using extrusion.

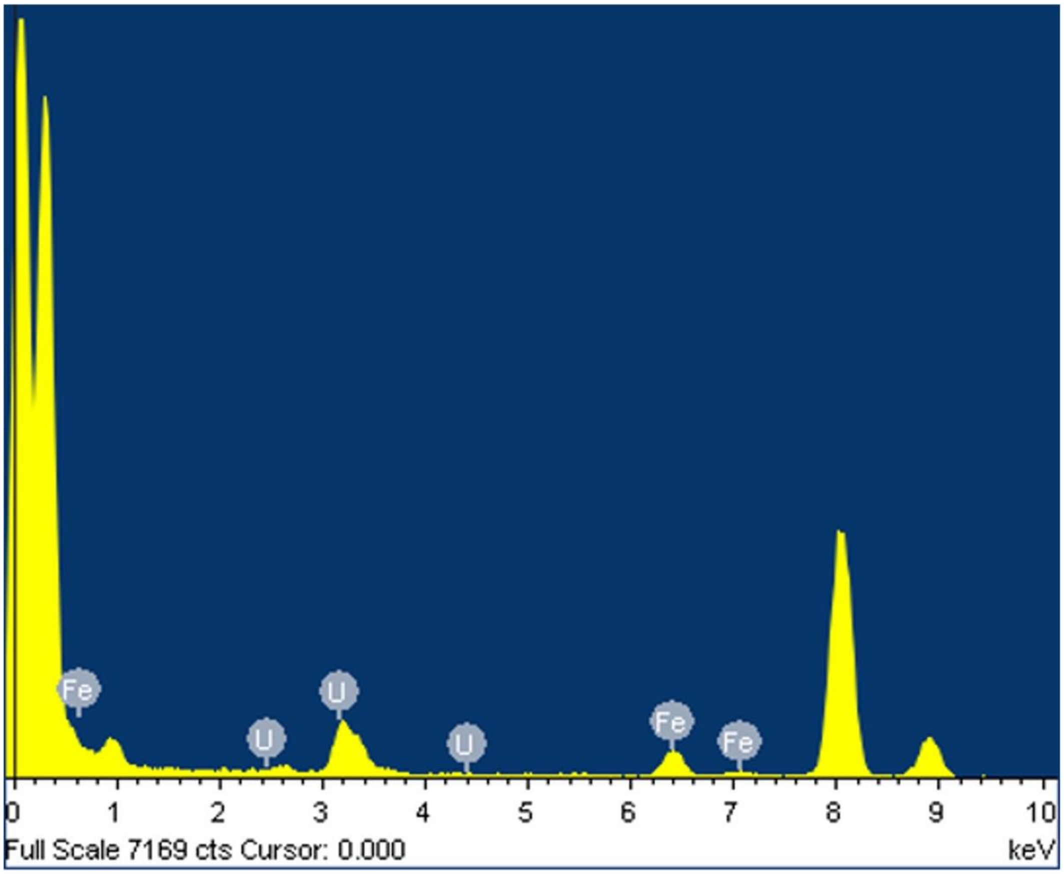

Element	Weight%	Atomic%
Fe K	17.78	47.96
U M	82.22	52.04
Totals	100.00	

Figure S6: Corresponding EDX spectrum for uranyl acetate-stained Exo:PB in Figure 2F. The table represents weight and atomic percent of iron and uranium detected.

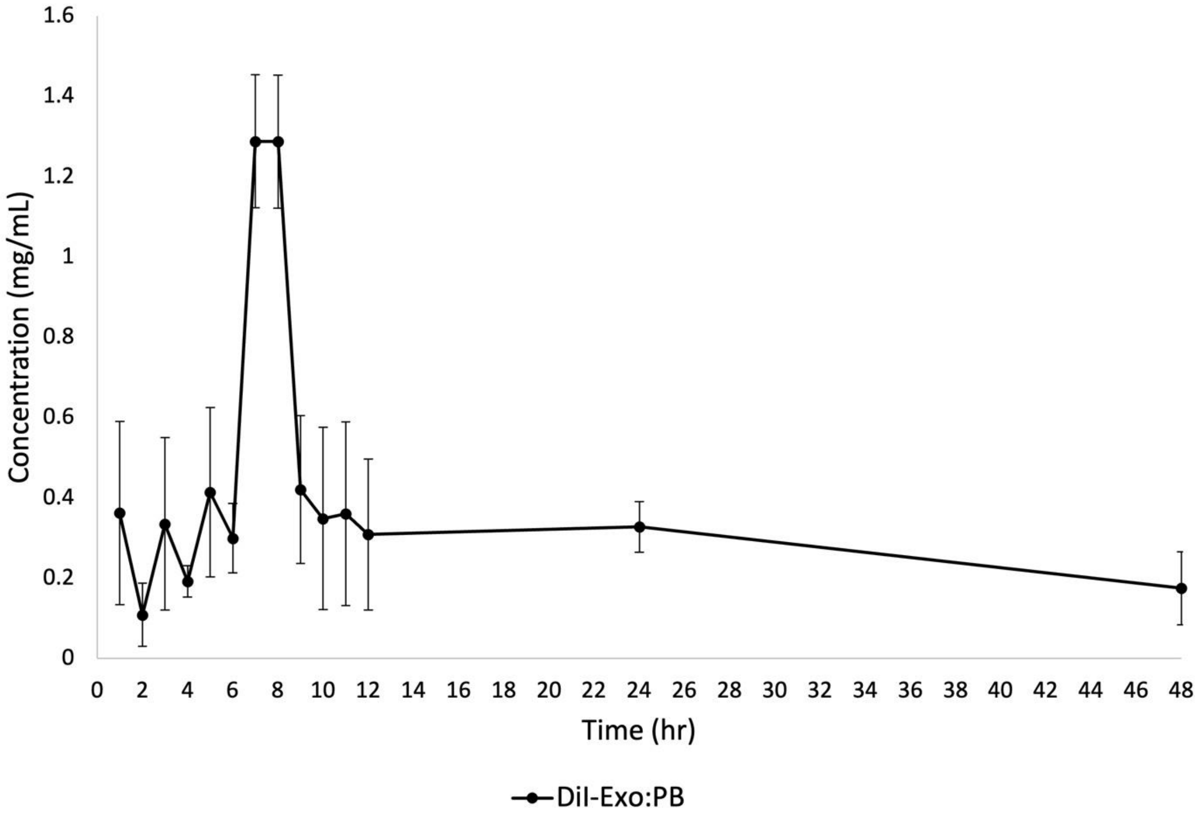

Figure S7: Cell uptake kinetics of DiI-Exo:PB within U-87 cells after 1, 2, 3, 4, 5, 6, 7, 8, 9, 10, 11, 12, 24, and 48 hours of incubation.

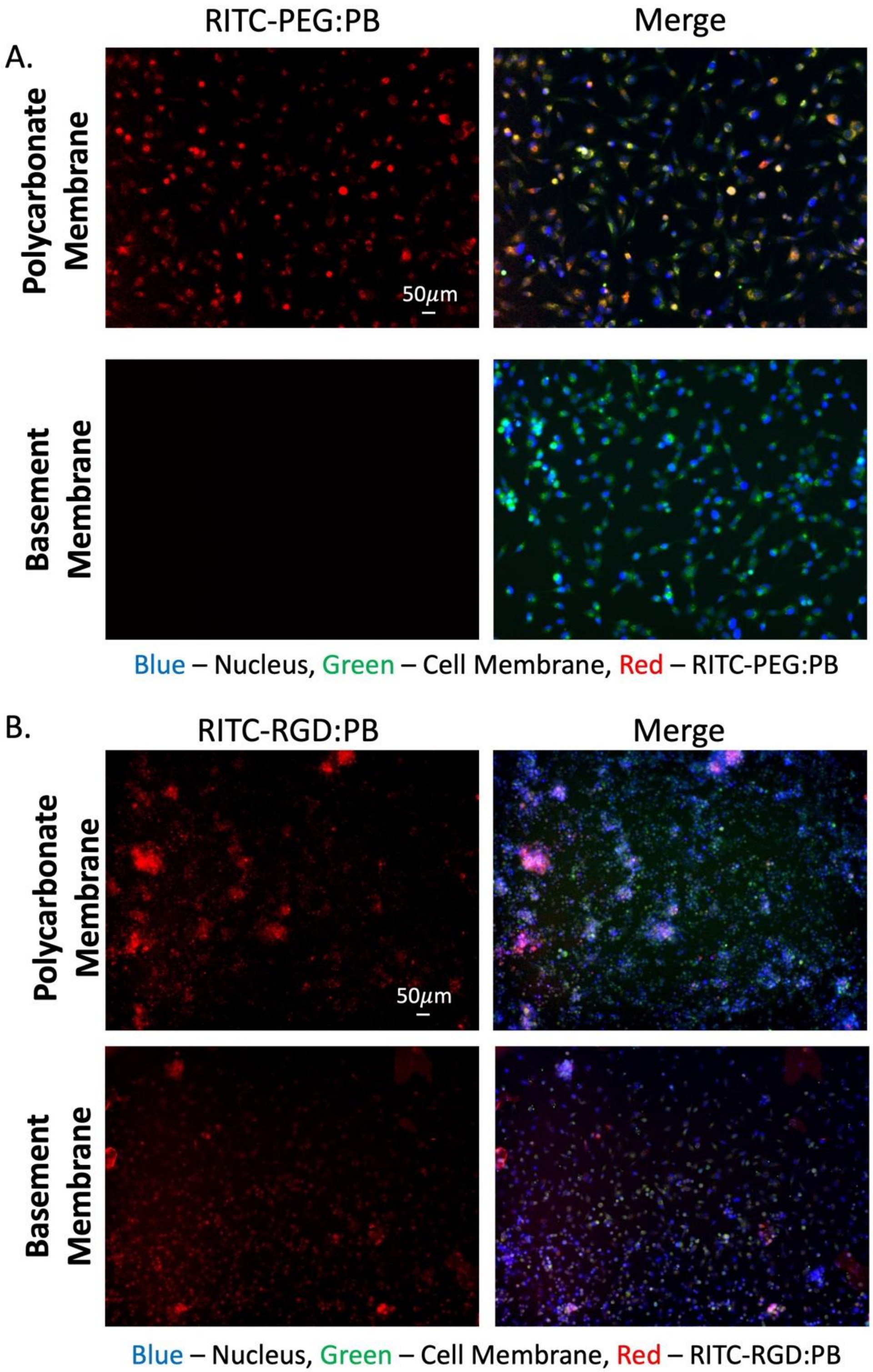

Figure S8: *In vitro* BBB cellular uptake within U-87 cells. (A) RITC-PEG:PB and (B) RITC-RGD:PB. Red = particle, Green = cell membrane, Blue = cell nucleus. Scale bar = 50 μm.

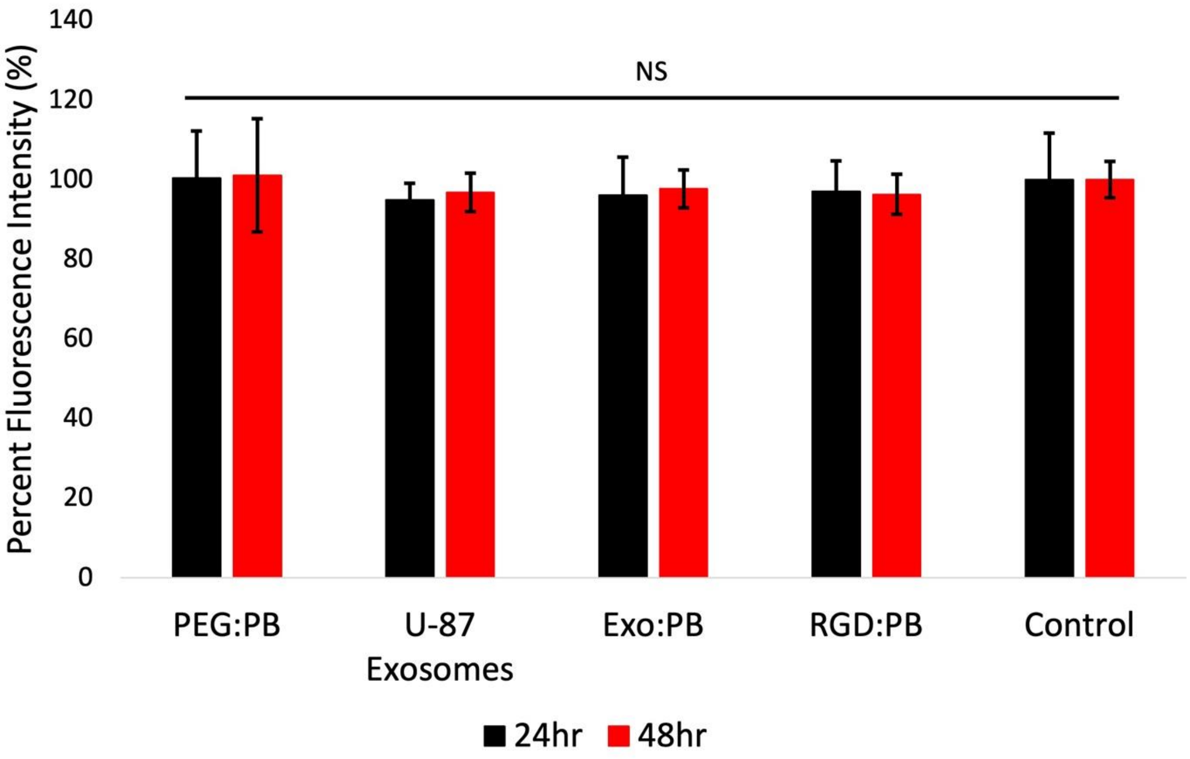

Figure S9: Possible Tumorgenesis Effects. In vitro cell growth monitored in U-87 cells for 24 (Black) and 48 (Red) hours after incubation with PEG:PB, U-87 exosomes, Exo:PB, RGD:PB, or nothing. NS = no significance.

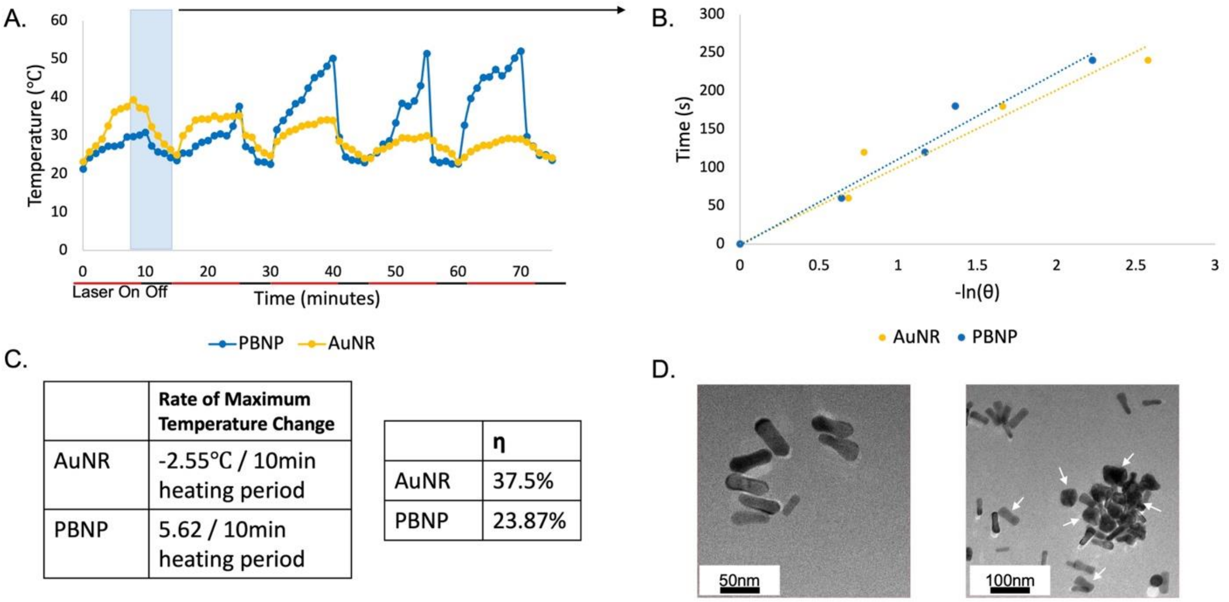

	Rate of Maximum Temperature Change
AuNR	-2.55°C / 10min heating period
PBNP	5.62 / 10min heating period

	η
AuNR	37.5%
PBNP	23.87%

Figure S10: AuNR Temperature profile comparison. (A) Temperature gradient of PBNP (blue) and AuNR (yellow) when exposed to an 808 nm laser (red = laser on, black = off). Table expresses rate of temperature change over each 10-minute heating period. (B) Linear correlation of the cooling curve in Figure S4A for PBNP (blue) and AuNR (yellow). (C) Change of maximum temperature change over each cycle from Figure S4A and calculated photothermal conversion efficiency (η) for AuNR and PBNPs. (D) TEM images of (i.) before and (ii.) after laser exposure of AuNR. Scale bar is 50 nm (before) and 100 nm (after). White arrows indicate areas of morphological change.

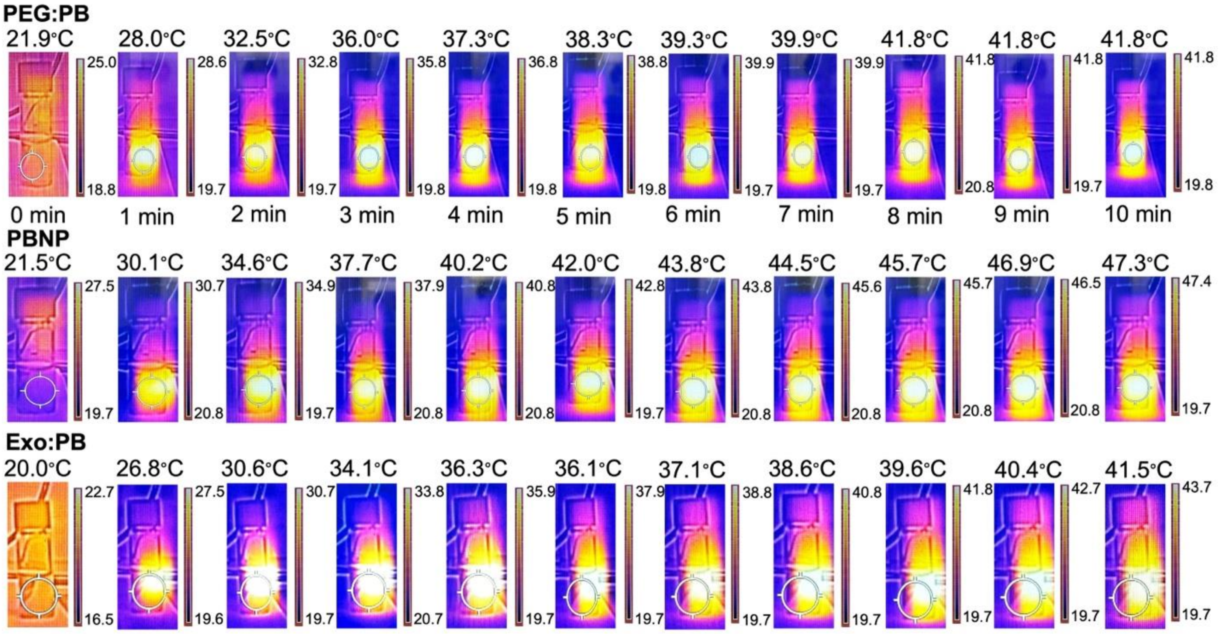

Figure S11: Thermal camera images of PBNP, PEG:PB, and Exo:PB during a 10-minute exposure to an 808 nm laser.

A.

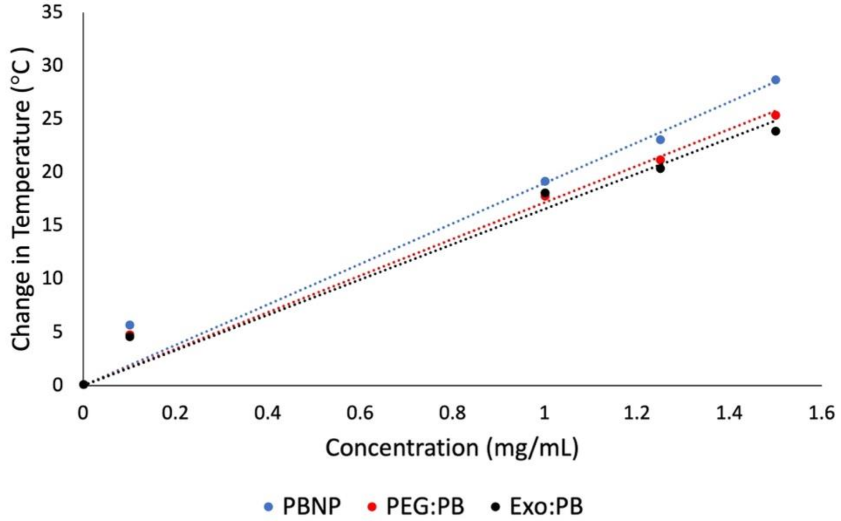

B.

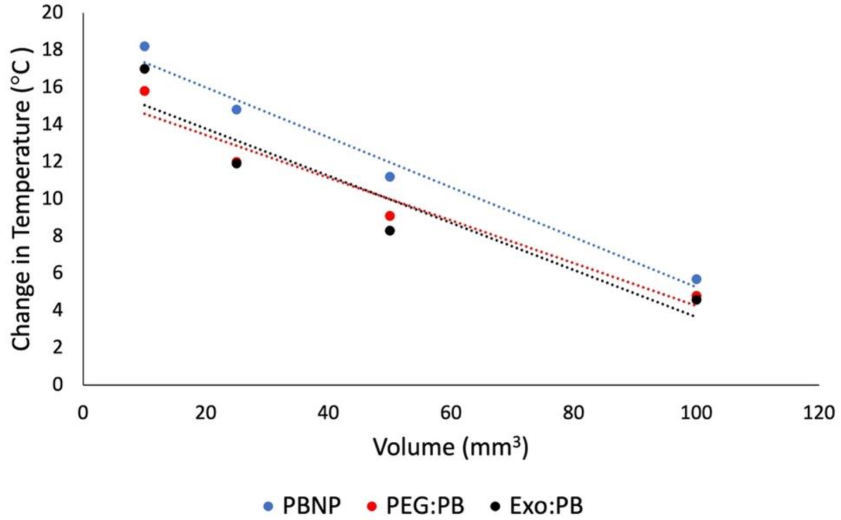

C.

	Concentration in Cell Pellet (mg/mL)	Calculated Temperature (∘C) Reached within Cells
PBNP	0.105 ± 0.0190	55.67
PEG:PB	0.103 ± 0.0034	52.73
Exo:PB	0.122 ± 0.0251	53.30

Figure S12: Maximum Temperature on Single Cell Level. (A) Concentration vs change in temperature and (B) volume vs change in temperature for PBNP (blue), PEG:PB (red), and Exo:PB (black). (C) Table of calculated particle concentrations within U-87 cell pellets from (A) and estimated maximum temperature reached with a single U-87 cell from (B). Final value was calculated using an assumed starting temperature of 37°C for the cells and an average size of a U-87 cell to be 4.6875×10^{-5} mm^3.

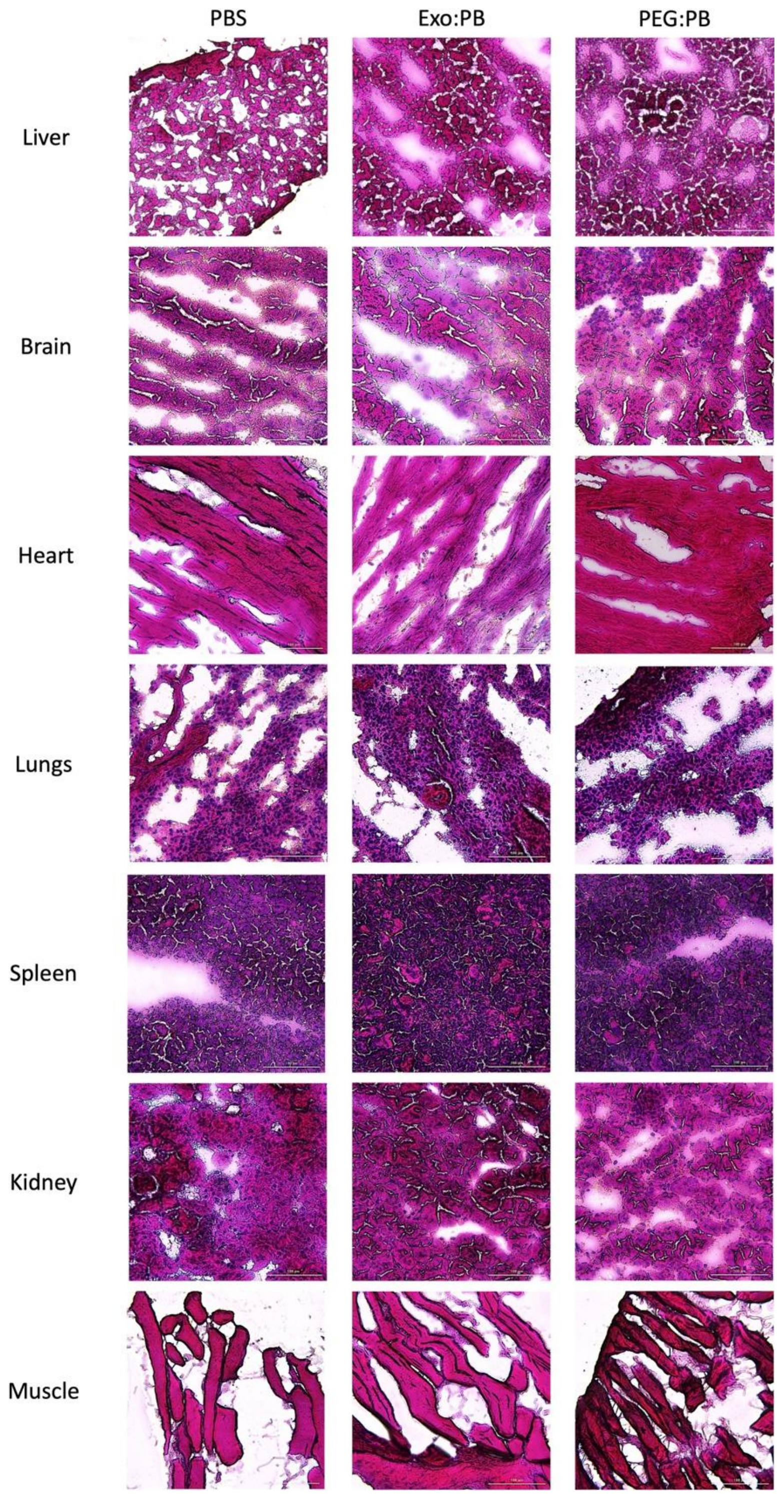

Figure S13: H&E staining of subcutaneous tumor bearing mouse tissue 24 hours after intravenous injection of PBS, Exo:PB, or PEG:PB.

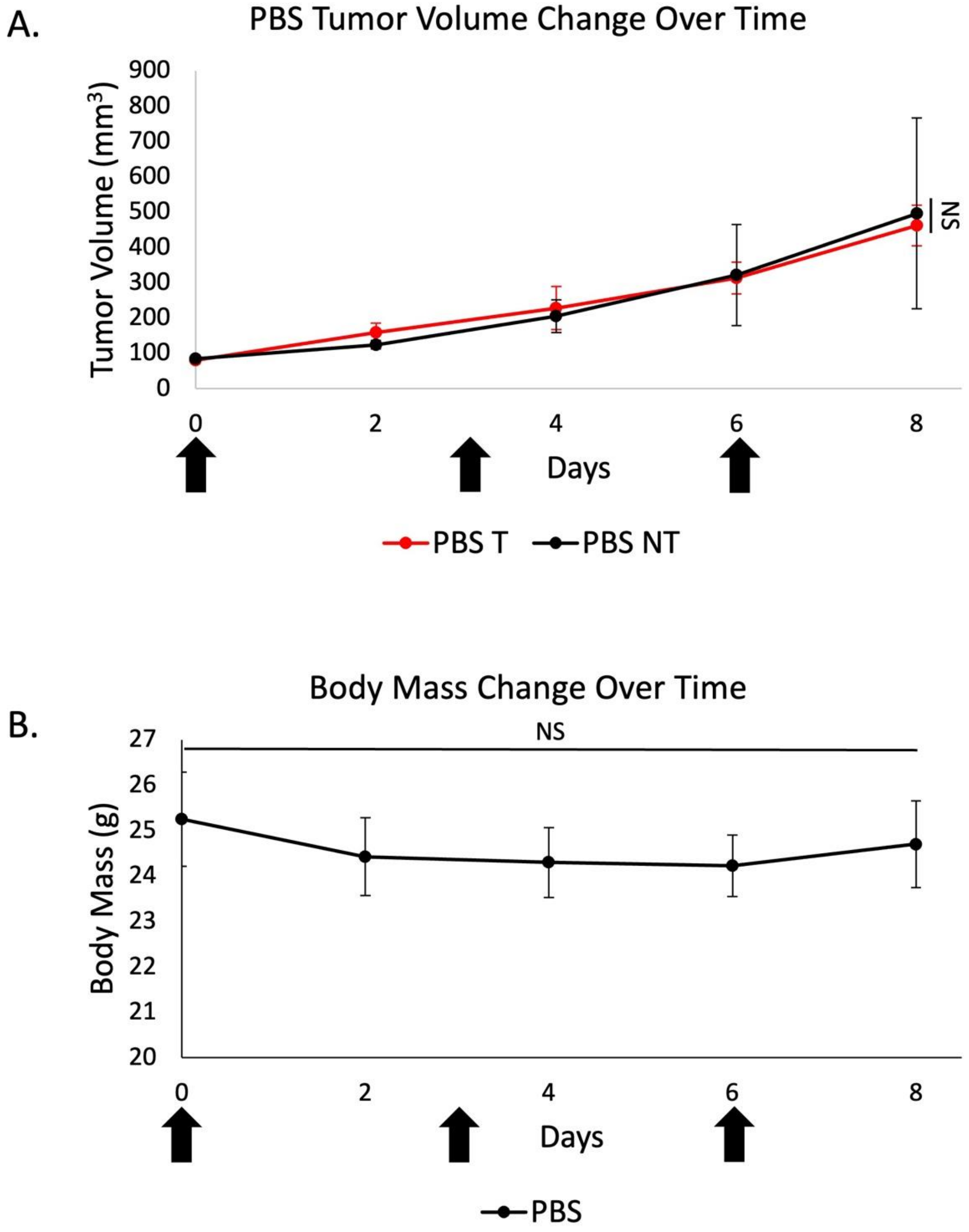

Figure S14: Size change in U-87 subcutaneous tumors after PBS intravenous injection. (A) Tumor size after PBS injection and laser treatment. Arrows indicates days in which injection and laser exposure occurred. (B) Body mass of mice during treatment period. Arrows indicate days in which injection and laser exposure occurred. N=3. NS = no significance.

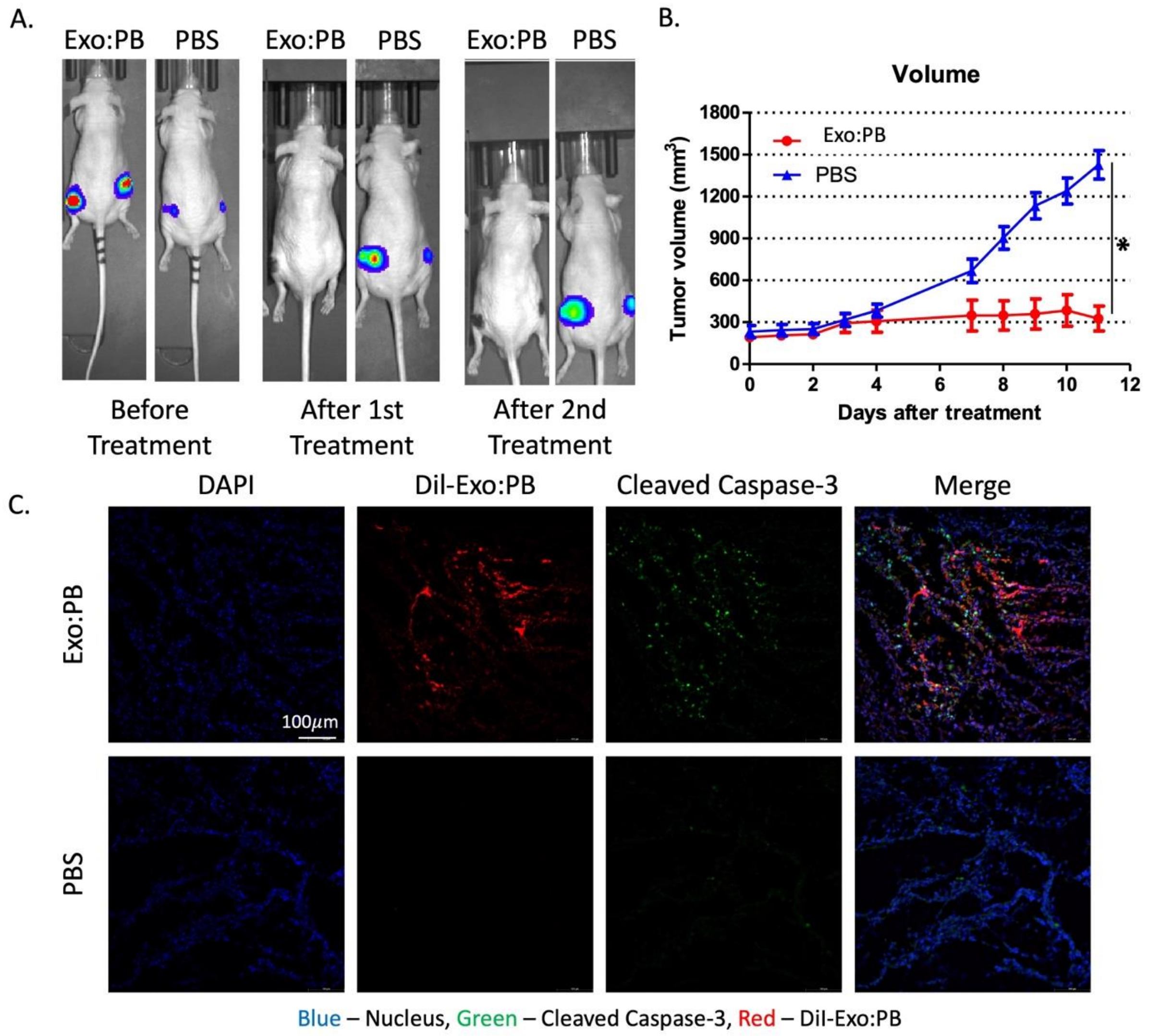

Figure S15: (A) IVIS bioluminescent tumor signal before and after first and second treatments with PBS or Exo:PB and laser. (B) Normalized quantitative tumor volume in mice before and after first and second treatments (*p<0.01). (C) Apoptotic assay results in tumor sections after photothermal treatment. Blue = cell nucleus, Red = DiI-Exo:PB, Green = Cleaved Caspase-3, Scale = 100 μm.

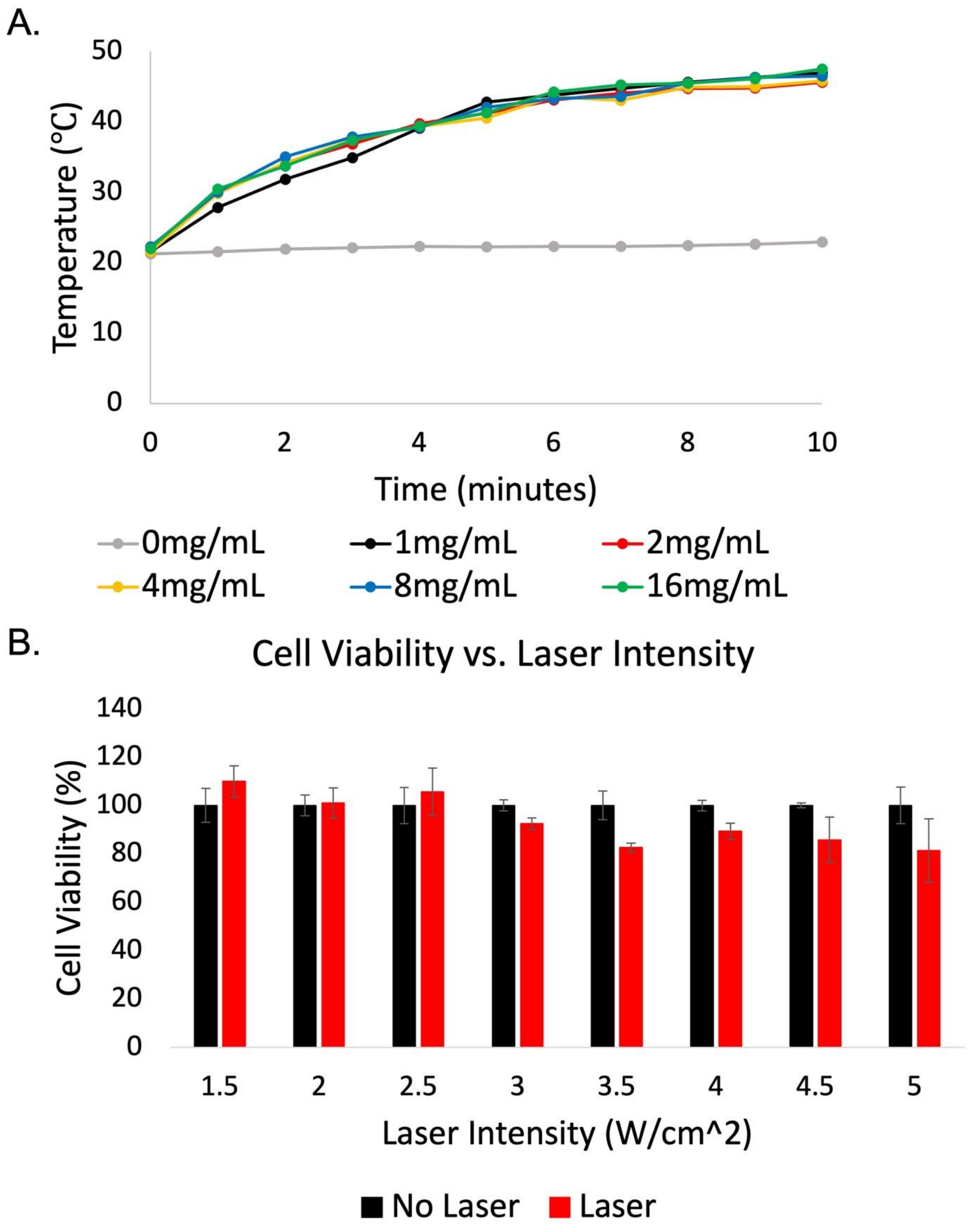

Figure S16: (A) Photothermal Intensity of PBNPs based on mg/mL concentration. (B) MTT assay results of U-87 cells exposed to increased laser intensity with no PBNPs present.

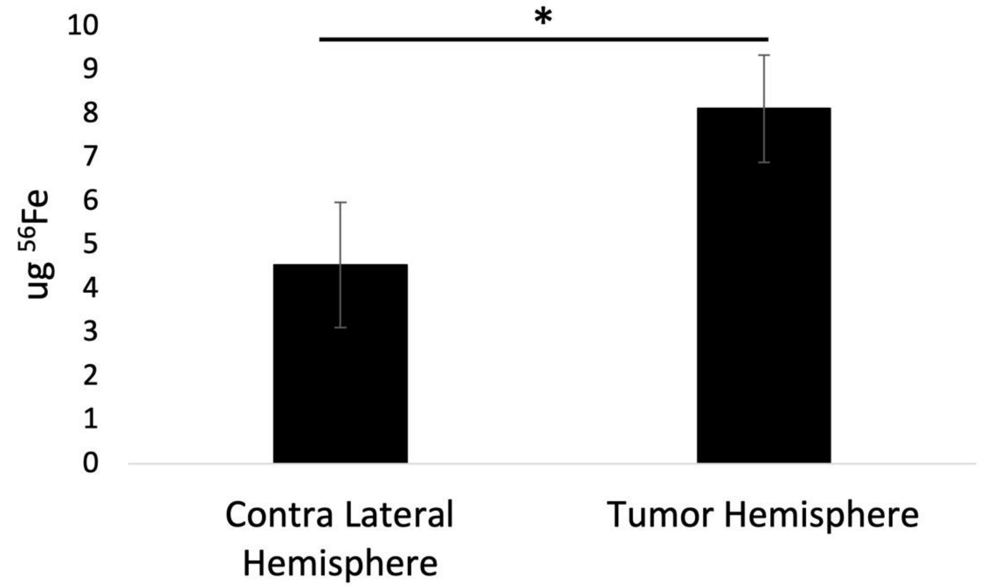

Figure S17: ICP ^{56}Fe microgram quantification in the tumor hemisphere vs contra lateral hemisphere. N=3, *$p<0.05$.

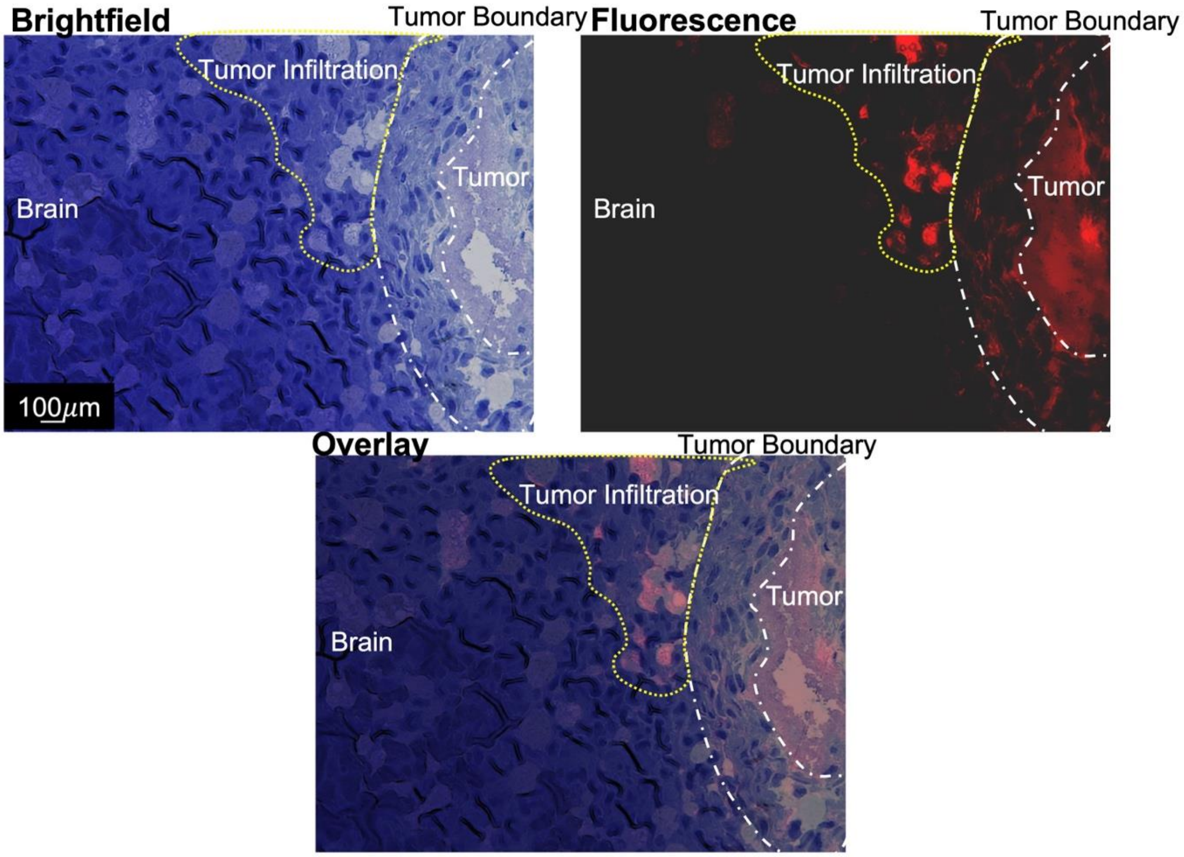

Figure S18: Corresponding brightfield and overlay fluorescence images to the BioTEM images presented in Figure 6E. White dotted line indicates tumor boundary (blood-brain tumor boundary), yellow dotted line indicates tumor cell infiltration into healthy tissue. Red = DiI-Exo:PB. Scale bar = 100 μm.

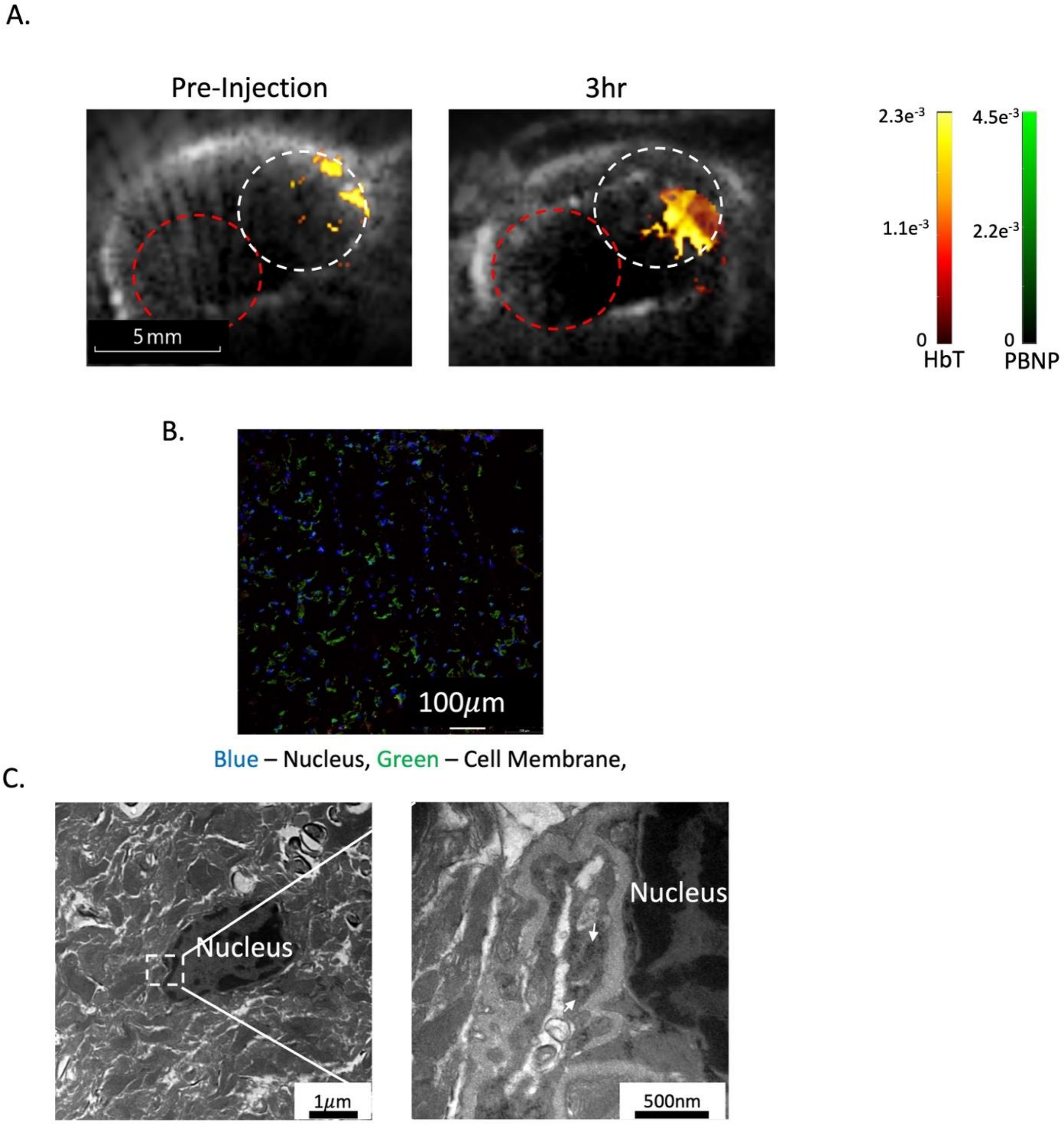

Figure S19: PEG:PB orthotopic glioblastoma PAI and *ex vivo* comparison. (A) PA images of U-87 orthotopic brain model mice after intravenous injection of PEG:PB to determine particle accumulation within the brain tumor region. Red = Total Hemoglobin Concentration, Green = PBNP. White Circle = Tumor Hemisphere, Red Circle = Contra Lateral Hemisphere. Scale bar = 5 mm. (B) Brain tissue section with overlay cell nucleus (blue), cancer marker Ki67 (green), and RITC-PEG:PB (red). Scale bar = 100 μm. (C) BioTEM of brain tumor regions after intravenous injection of PEG:PB. Yellow arrows indicate PEG:PB. Scale bar = 1 μm (5,000 X) and 500 nm (15,000 X).

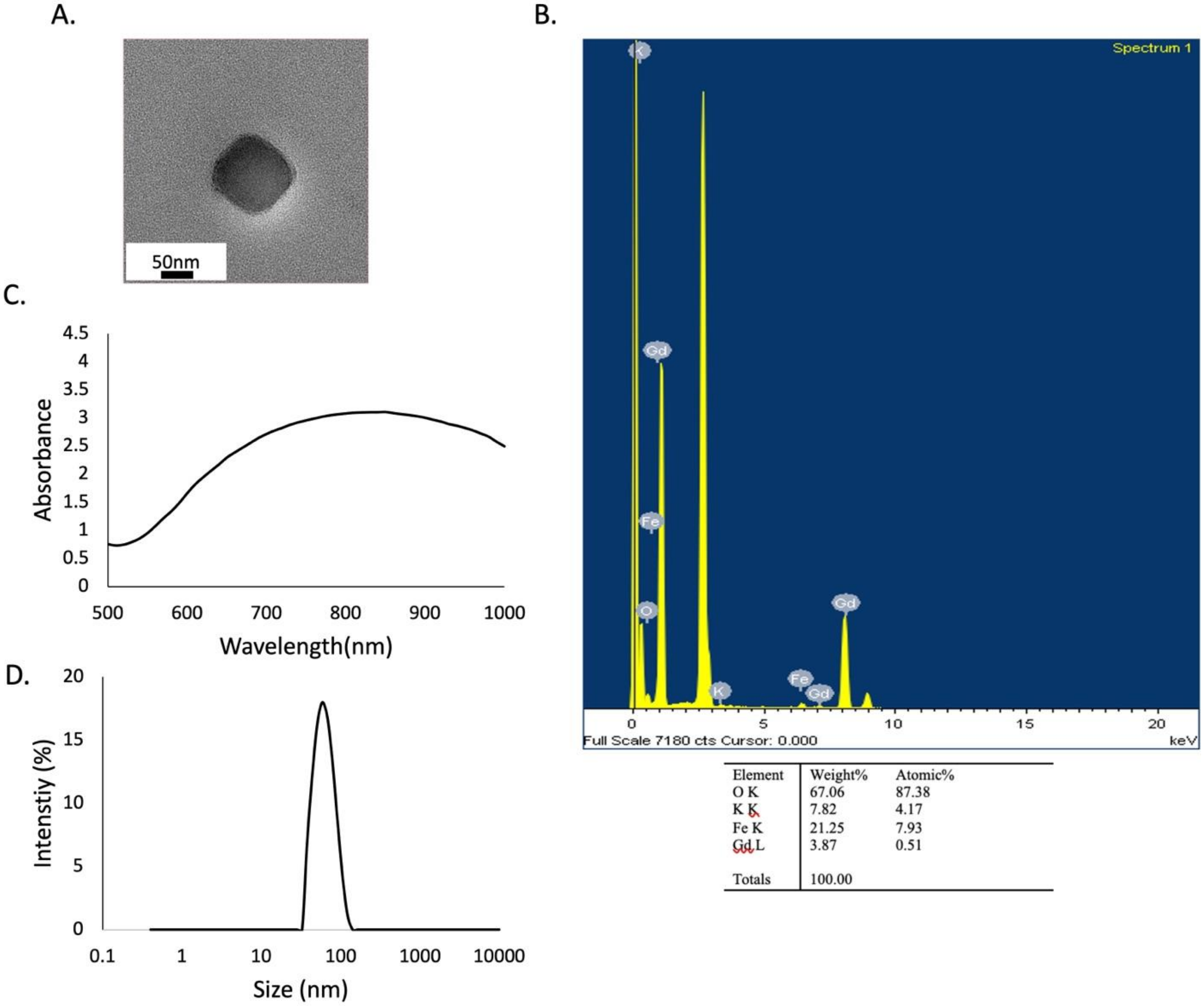

Element	Weight%	Atomic%
O K	67.06	87.38
K K	7.82	4.17
Fe K	21.25	7.93
Gd L	3.87	0.51
Totals	100.00	

Figure S20: Characterization of Gd:PB particles. (A) Gd:PB TEM image. Scale = 50 nm with corresponding EDS spectrum(B). (C) Absorbance and (D) hydrodymanic diameter of Gd:PB particles.

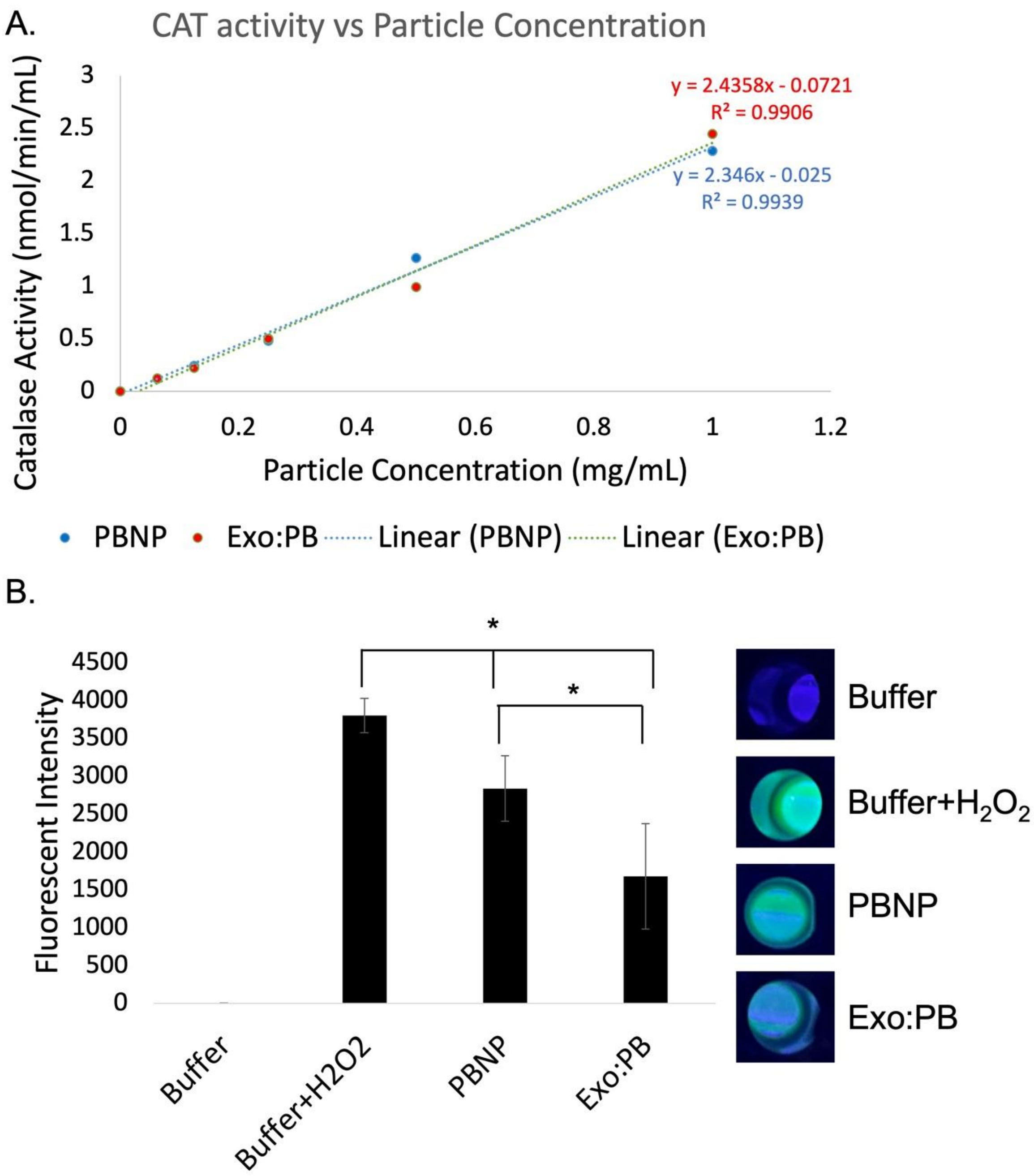

Figure S21: (A) PBNP (blue) and Exo:PB (red) catalase activity based on increasing particle concentration. (B) Fluorescent hydrogen peroxide degradation assay results (*p<0.05).

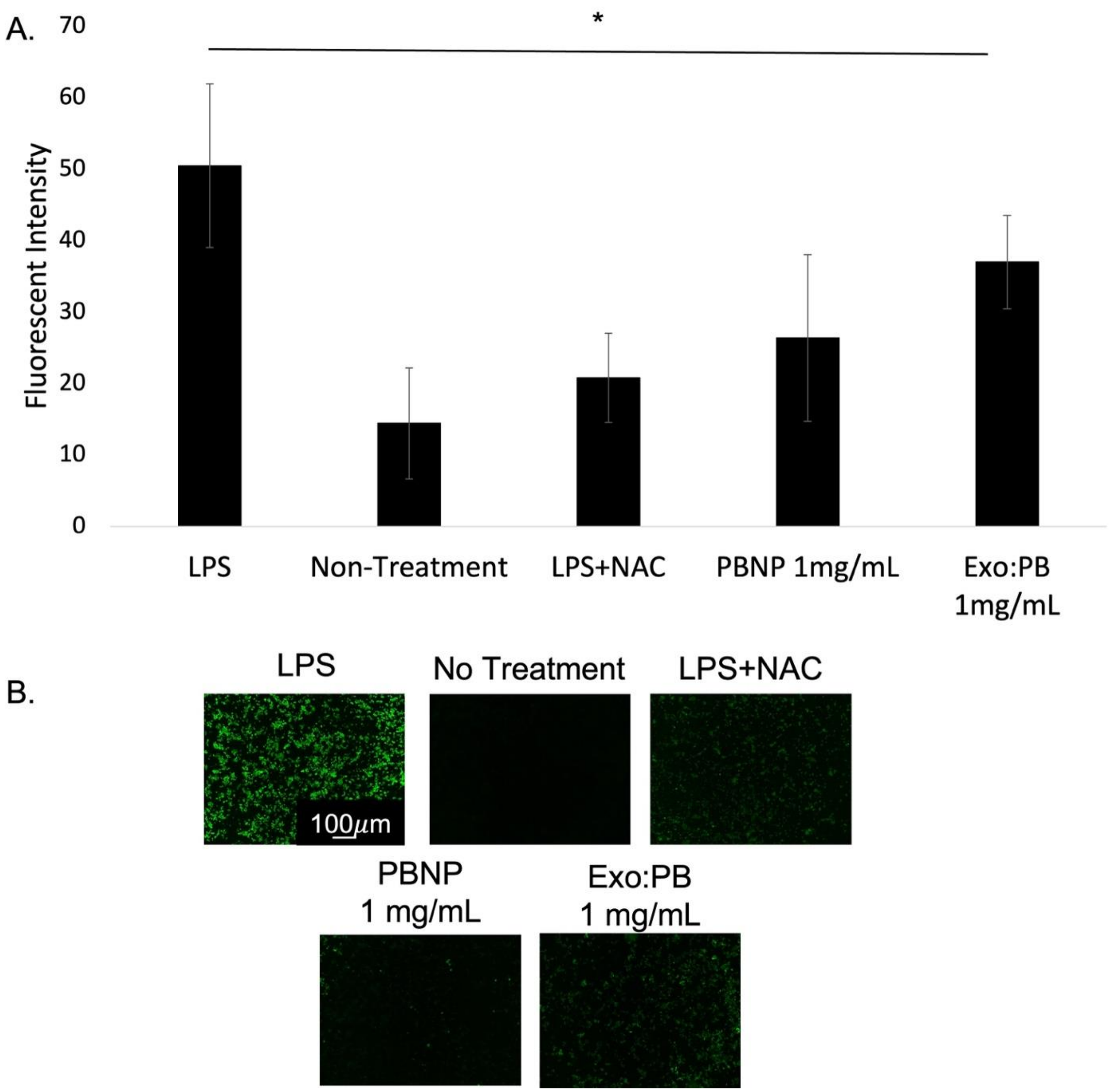

Figure S22: (A) Quantitative DCFDA assay results using Raw 264.7 cells and 1 mg/mL of PBNP or Exo:PB (*$p<0.05$). (B) Representative fluorescent images from wells from the DCFDA assay. Scale bar = 100 μm.

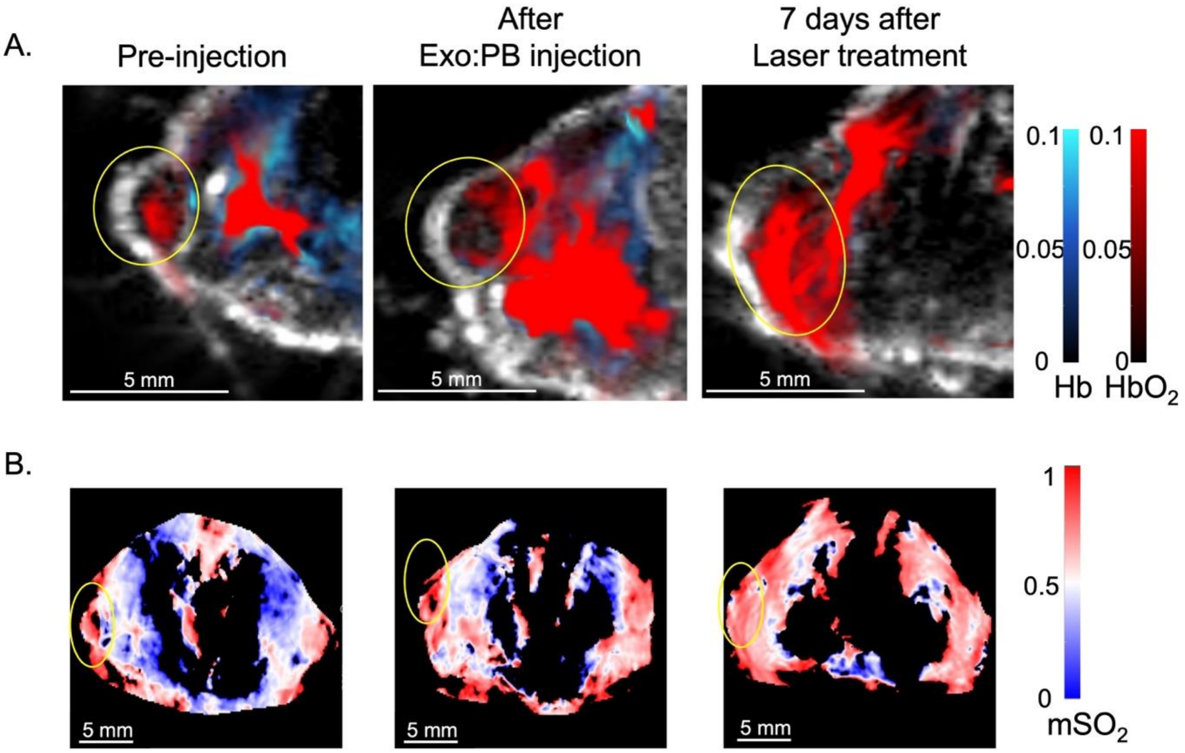

Figure S23: (A) Blood oxygen overlay with PA images and (B) mSO_2 PA images of subcutaneous U-87 tumor models before and after Exo:PB injection and laser treatment. Red = hemoglobin, Blue = deoxyhemoglobin, Scale bar = 5 mm. Yellow circle indicates tumor regions.
